# From Engraftment to Vision: Defining Evidence of Functional Success in Retinal Cell Replacement Therapy

**DOI:** 10.3390/cells15201840

**Published:** 2026-10-10

**Authors:** Georgios D. Panos, Theo Empeslidis, Eleftherios Chatzimichail, Lorenzo Motta, Zisis Gatzioufas, Winfried Amoaku

**Affiliations:** 1 Division of Ophthalmology and Visual Sciences, School of Medicine, University of Nottingham, Nottingham NG7 2UH, UK; 2 First Department of Ophthalmology, AHEPA University Hospital, School of Medicine, Aristotle University of Thessaloniki, 54636 Thessaloniki, Greece; 3 Vantage Biosciences Ltd., London SW1Y 5ES, UK; 4 Department of Ophthalmology, University Hospital of Basel, 4031 Basel, Switzerland; 5 Ophthalmology Unit, Department of Neuroscience, University of Padua, 35122 Padua, Italy

**Keywords:** retinal cell replacement, retinal pigment epithelium, photoreceptor transplantation, retinal organoid, retinal ganglion cell, functional integration, material transfer, causal inference

## Abstract

Retinal graft survival and post-treatment visual improvement do not, alone or together, demonstrate functional cell replacement. Apparent benefit may reflect material transfer, trophic rescue, residual host function or procedural and measurement effects. This critical narrative review uses a structured PubMed search covering January 2020 to August 2026, supplemented by seminal studies, to assess retinal pigment epithelium (RPE), photoreceptor, retinal sheet or organoid, and retinal ganglion cell (RGC) transplantation. We separate mechanistic attainment, downstream outcomes and claim-specific causal confidence. Authentic RPE replacement requires donor epithelial function across the host interface, without new synapses. Photoreceptor replacement requires donor phototransduction and transmission to host retinal circuitry; composite grafts additionally require identification of the active donor components. RGC replacement requires both retinal afferent integration and functional reconnection with appropriate central targets. Selected photoreceptor studies demonstrate persuasive local physiological coupling, but durability, donor necessity and generalisability remain incompletely established. Human evidence principally supports feasibility, safety and graft-associated anatomy, rather than convincingly donor-mediated visual restoration. We propose a non-linear evidence framework in which outcomes cannot substitute for secure donor provenance, cell-specific function or host–graft coupling. Spatial concordance, durability, controls, disease-model relevance and experimental unit should qualify every claim.

## 1. Introduction

Retinal cell replacement could address irreversible cell loss, but producing a retinal cell product and detecting graft-associated tissue do not establish that donor cells have restored the missing function. The relevant questions are whether living donor cells perform their intended physiological role, interact with recipient tissue and contribute causally to retinal or visual recovery [1,2,3,4,5].

Common endpoints answer different parts of this problem. Imaging can document graft location, electrophysiology can identify retinal or pathway activity, and behaviour or clinical testing can demonstrate benefit. Their interpretation depends on donor identity, residual host function, spatial resolution and experimental controls [6,7,8]. Photoreceptor transplantation illustrates the consequences of overlooking these distinctions: donor-associated cytoplasmic labels can enter host cells, so apparently integrated reporter-positive cells need not be donor neurons [9,10,11,12]. Retinal pigment epithelium (RPE) pigmentation and molecular maturation likewise differ from donor epithelial function [13,14,15,16]. Selected photoreceptor studies provide persuasive local physiological coupling [17,18,19], whereas light responsiveness in transplanted retinal ganglion cells (RGCs) addresses retinal input without establishing functional reconnection to the brain [20,21].

This critical narrative review evaluates RPE, photoreceptor, retinal sheet or organoid, and RGC transplantation. Its primary purpose is to provide a conceptual framework for interpreting and reporting existing evidence. A secondary purpose is to identify the experiments needed to support a specified replacement claim. The framework is not a validated scoring instrument, a regulatory standard or a mandatory sequence for progression to clinical testing. It separates mechanistic evidence, downstream outcomes and causal confidence, while allowing the biological requirements to differ between epithelial, single-neuron and composite tissue products. Throughout, an unmeasured mechanism is distinguished from a mechanism tested and contradicted. Therapeutic benefit can remain valuable when its cellular basis is unresolved.

## 2. Review Methodology

We undertook a critical narrative review with a structured PubMed search, rather than a registered systematic review or quantitative meta-analysis. Searches were last run on 31 August 2026 and covered RPE replacement, photoreceptor transplantation, retinal sheet or organoid, and RGC transplantation. The contemporary interval was 1 January 2020 to 31 August 2026, without a language restriction at retrieval. Seminal earlier studies and mechanistic reports were added through targeted PubMed searches and reference lists. The original search strings, retrieval counts and detailed extraction fields are retained in Supplementary Section S1.

Selection was purposive: studies were included when they informed product competence, donor identity, localisation, donor function, host–graft coupling, physiological or visual outcomes, or an alternative explanation for rescue. Eligible products included stem- or progenitor-derived RPE suspensions, strips, monolayers and supported constructs; photoreceptor precursors and differentiated rods or cones; organoid-derived cells and retinal sheets; and RGC products. Predominantly paracrine stromal-cell treatments, extracellular vesicles, endogenous reprogramming, gene therapy, optogenetics and prostheses were considered only where relevant to interpretation. Negative and null findings were eligible.

Citation identity was checked against PubMed, DOI records, publishers or supplied primary text. Bibliographic verification was distinguished from access to methods and results. The analytical corpus comprised 79 studies: 71 were assessed at the full-text or methods level and eight remained partial-primary or abstract-level. Source-limited records were not used to infer unreported denominators or controls. Extraction covered product and recipient characteristics, follow-up, mechanistic and outcome evidence, causal controls, experimental units and methodological limitations. The framework was developed iteratively against representative studies.

Interpretation was restricted to evidence supported by the primary source. PubMed-only retrieval and purposive selection may have omitted relevant studies; the review does not claim exhaustive systematic coverage. Heterogeneous products, models and claims also preclude a defensible common numerical risk-of-bias score. Causal confidence was therefore assessed for individual claims. The proposed categories have not been validated as a reproducible measurement scale and require independent evaluation before any standard-setting use.

## 3. Defining and Assessing Functional Replacement

### 3.1. Purpose and Terminology

Functional replacement means that living donor-origin cells occupy the required compartment, perform the intended function after transplantation and interact appropriately with host tissue. Survival, anatomical integration, functional rescue and visual restoration describe different observations (Table 1). Improved function after transplantation may support a treatment effect without identifying its mechanism. Conversely, authentic local donor function need not produce measurable whole-eye or behavioural benefit when the graft is small or the remaining pathway is severely damaged.

The framework provides a structured language for these distinctions. It can guide study design by making the intended claim and its missing evidence explicit, but does not determine product approval, regulatory compliance or readiness for a clinical trial. Mechanistic uncertainty should narrow the claim; it should not automatically negate a therapeutic observation or preclude a safety study. Decisions about clinical progression also require product, safety and trial considerations beyond this review.

### 3.2. Independent Evidence Domains and Assessment Status

**Figure 1:**
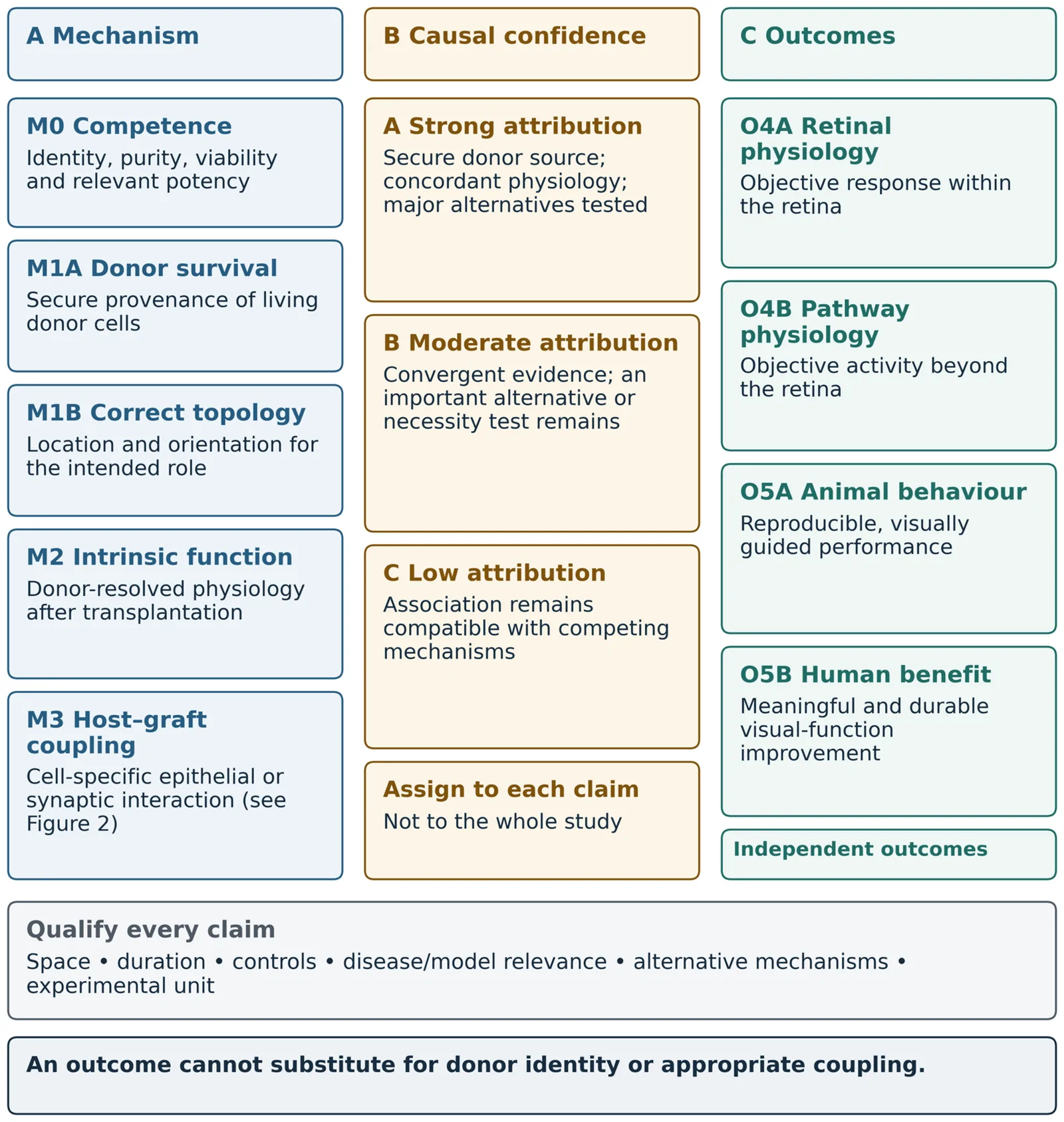
Independent dimensions of retinal cell-replacement evidence: mechanism, claim-specific causal confidence and outcomes. These are not sequential ranks or an additive score. Each domain is recorded as not assessed, not met, supportive or demonstrated, with the reason stated. An unmet evidential criterion does not necessarily constitute evidence against replacement.

Mechanistic attainment comprises product identity and competence (M0), donor provenance and survival (M1A), appropriate topology (M1B), intrinsic donor function after transplantation (M2), and cell-specific host–graft coupling (M3). Retinal physiology (O4A), downstream pathway physiology (O4B), animal visual behaviour (O5A) and human visual benefit (O5B) are recorded separately. These coordinates are not sequential ranks: observing O5 does not validate unmeasured M2 or M3. Pre-transplant potency, marker expression and morphological maturation cannot establish post-transplant physiological performance [13,14,22]. Figure 1 shows this architecture; Table 2 specifies minimum evidential criteria and practical limitations.

Each domain is recorded as not assessed, not met, supportive or demonstrated, with a reason. “Not assessed” means that no relevant test was performed. “Not met” means that an assessed criterion was not satisfied; the reason must distinguish inadequate or inconclusive evidence from an adequately measured negative result. “Supportive” denotes relevant evidence that leaves a material inferential gap; “demonstrated” denotes evidence meeting the stated criterion within the tested conditions. These labels describe the evidence available, not certainty that replacement occurred or failed.

Evidence against replacement requires a discriminating finding inconsistent with the specified mechanism under conditions in which the assay could detect it. Its scope remains local to the function, recipient and time tested. For example, a negative full-field electroretinogram (ERG) does not exclude a small functioning graft, and an unmeasured central projection does not demonstrate that no axon reached the brain. An absent donor-identity assay leaves provenance unresolved. Explicit reasons are therefore more informative than a label alone, and no domain is automatically inferred from another.

### 3.3. Cell-Specific Implementation

For RPE, M2 asks whether donor cells execute an epithelial task; M3 asks whether that task functions across the host interface. These domains can overlap biologically and experimentally. Donor-resolved uptake and processing of host outer segments, for example, may inform both. Such evidence should be described once and linked to both domains without being counted as independent confirmation. The framework does not require separate assays solely to preserve the distinction. Polarity or a static outer-segment inclusion remains supportive when physiological processing is unmeasured, and no synaptic criterion applies.

For photoreceptors, intrinsic light detection and signal transmission to host retina are distinguishable processes. M2 requires evidence attributable to donor phototransduction; M3-PR requires physiological transfer into host circuitry. Composite sheets require the claimed functional unit to be named: the whole product, a specified donor cell class, or a reconstructed circuit. Internal donor–donor activity is graft maturation. Physiological crossing of an identified donor–host boundary supports coupling; when the participating donor cell classes remain unresolved, the claim should remain composite rather than cell-specific. A downstream response alone does not establish even composite coupling.

**Figure 2:**
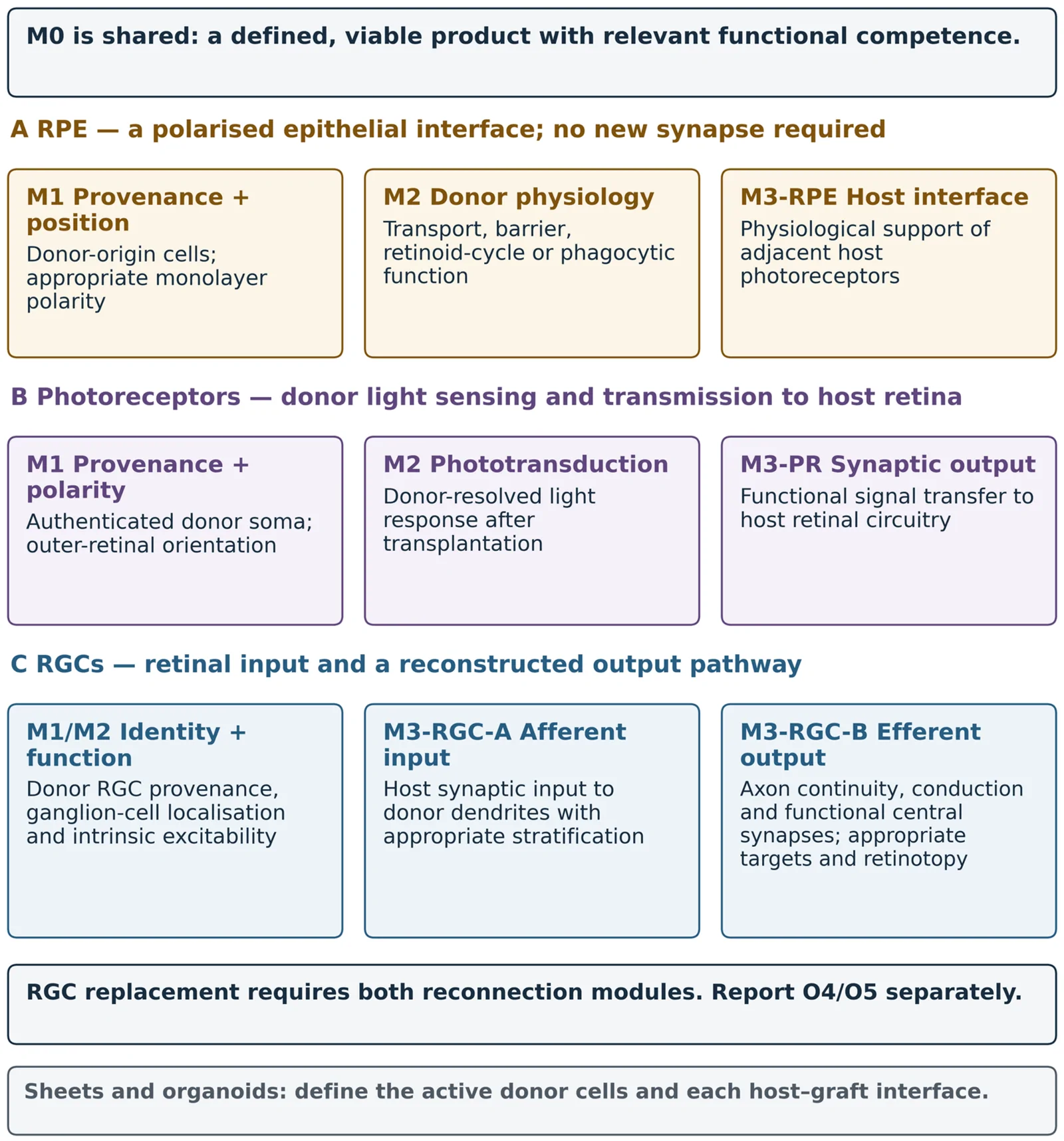
Cell-specific requirements for RPE, photoreceptor and RGC replacement. RPE M2 and M3 may be informed by the same functional interface experiment without double-counting. RGC afferent and efferent interfaces are separate requirements. Sheets and organoids are assessed at the stated product, cell-class or circuit resolution; internal graft activity is distinct from host coupling.

RGC replacement requires two interfaces. M3-RGC-A concerns input from host retinal circuits to donor dendrites; M3-RGC-B concerns donor axonal output to appropriate central targets. Intrinsic excitability supports M2, whereas synaptically driven donor light responses support M3-RGC-A. Evidence for one interface cannot substitute for the other. Figure 2 summarises these biological differences.

### 3.4. Causal Confidence and Claim Boundaries

Grade A denotes strong attribution for a specified claim: secure provenance, spatially and physiologically linked evidence, adequate controls and direct testing of major alternatives. Grade B denotes convergent evidence with an important unresolved alternative or necessity test. Grade C denotes an association also plausibly explained by host preservation, material transfer, trophic action, procedural effects or uncontrolled change. These are interpretative categories, not numerical weights, and the same paper may support different grades for survival, coupling and visual benefit.

Every judgement should report spatial concordance, durability, control adequacy, disease-model relevance and alternative mechanisms, together with the independent experimental unit. Cells, electrodes, image regions and repeated measurements nested within recipients do not constitute independent treatment replicates. These considerations qualify the claim and cannot compensate arithmetically for a missing mechanistic link.

Within this proposed terminology, a functional replacement claim requires a competent product, secure donor provenance, suitable topology, demonstrated relevant donor function and physiological host coupling, with at least moderate causal confidence. This is an interpretative convention rather than a validated threshold. RPE overlap and composite circuits are handled as described above, not forced into separate sequential achievements. Physiological or visual restoration additionally requires the corresponding outcome and credible attribution. Donor-selective loss of function can strengthen that attribution, but its absence does not erase convergent evidence or constitute evidence that replacement failed. Stronger mechanistic tests and feasible clinical approaches are considered in Section 10.

## 4. RPE Replacement

### 4.1. Epithelial Function and Product Format

RPE replacement reconstructs a polarised interface between the choroid and surviving photoreceptors. Relevant functions include barrier regulation, vectorial transport, ion and fluid homeostasis, retinoid recycling and outer-segment processing. Local replacement may therefore be biologically meaningful without an immediate acuity gain [2,3,6]. Its physiological consequence can be preservation of host photoreceptors: preservation is compatible with successful RPE replacement, but requires evidence of donor epithelial function to distinguish it from non-specific trophic rescue.

Suspensions, strips and supported monolayers face different delivery constraints. Dispersed cells must attach and repolarise on a diseased substrate; intact constructs preserve junctions but can fold, detach or cause insertion trauma. Format does not determine evidence status, and scaffold visibility does not establish cell viability [13,23,24,25]. Carrier effects also require attention. Acellular scaffolds caused local degeneration in minipigs, fibrin-supported porcine grafts lacked a concurrent acellular-fibrin arm, and microgel-supported grafts in RCS rats combined uncertain lineage with possible trophic effects of both carriers and cells [26,27,28]. These findings support product-associated effects without isolating epithelial replacement. Additional design details are retained in Supplementary Section S4.

### 4.2. Donor Identity and Functional Evidence

Pigmentation and optical coherence tomography (OCT) thickness can reflect donor RPE, host migration or hyperplasia, macrophages or persistent implant material. Reciprocal donor–recipient human leucocyte antigen (HLA) staining in a two-year human postmortem specimen provided substantially stronger provenance: donor RPE persisted on the parylene membrane with RPE65/BEST1 expression and apical Na^+^/K^+^-ATPase [15]. Five-year patch imaging, by contrast, could not identify the living cellular source [29]. In the nine-year Stargardt postmortem case, graft-site pigmented RPE-like tissue was real, but donor-specific nuclear or genomic evidence was unavailable and the untreated eye contained host RPE hyperplasia [16]. Donor persistence was therefore unresolved, rather than disproved.

Molecular maturation and epithelial physiology must also be separated. Transplanted RPE can approach an adult transcriptomic phenotype, but this does not directly measure transport or retinoid cycling [14]. Rhodopsin-positive inclusions in donor-proven cells are consistent with uptake of host outer segments and provide supportive interface evidence [13,15,24,30]. A static inclusion does not establish sustained ingestion, complete lysosomal processing or the other functions of RPE. The same observation should not be presented as separate confirmation of M2 and M3.

A minimum functional claim should identify the donor cells, their orientation and at least one relevant epithelial process operating at the host interface. The claim should name the function demonstrated rather than imply restoration of the complete RPE repertoire. Pulse-chase processing, donor-resolved retinoid tracing, directional transport or barrier measurements are candidate approaches; none is universally required. Spatially matched host support strengthens interpretation but cannot alone identify the responsible donor process. Donor-specific functional perturbation would provide stronger causal discrimination where technically feasible. Direct measurements in human recipients remain difficult, making matched preclinical evidence and rare explant or postmortem specimens particularly informative.

### 4.3. Preclinical Rescue and Durability

The Royal College of Surgeons (RCS) rat is useful because its MERTK-deficient RPE cannot perform normal outer-segment phagocytosis, but it also creates a major attribution problem. Transplantation is commonly performed while host photoreceptors and inner retinal circuitry remain available. A larger electroretinogram (ERG), superior-colliculus response or optokinetic threshold can therefore arise because donor cells, secreted factors or a carrier preserve residual host photoreceptors; it need not show reconstruction of an already absent epithelial-photoreceptor unit. Focal ERG over a pigmented graft region improved spatial concordance, as in a good manufacturing practice (GMP)-grade induced pluripotent stem cell (iPSC)-RPE study, yet the recorded signal still originated principally from host neural retina and the authors considered the mechanism uncertain [31]. Early treatment may demonstrate prevention of degeneration, which is clinically valuable, but should be labelled preservation rather than restoration.

Durability separates graft persistence from functional replacement. Nanocoated human embryonic stem cell (hESC)-derived RPE survived to 21 weeks in RCS rats, whereas the early ERG advantage was no longer recordable in either RPE-treated group at the terminal interval [23]. In a longer induced pluripotent stem cell (iPSC)-RPE monolayer study, donor RPE survived in seven of 15 eyes at 11 months and an intact monolayer remained in four; eight eyes had complete graft loss. Superior-colliculus responses were reported in five of eight OCT-selected eyes, without a relationship to the extent of donor survival, and optokinetic benefit present at four months was absent in all transplanted animals at 11 months [32]. These are not merely disappointing efficacy results. They show that M1, O4 and O5 can diverge over time and that a peak response cannot be used to infer terminal donor function.

Primate implantation demonstrates that preformed monolayers can retain polarity and take up host outer-segment material, although favourable structural results occur in subsets over short follow-up [13,24]. Human iPSC-RPE strips survived and expanded for up to six months in primate sites without significant preservation of outer-nuclear-layer thickness relative to laser-only regions; physiology was not tested [25]. Immune-evasion and ex vivo JAK-inhibition studies address graft survival rather than donor epithelial performance [33,34]. Healthy or acutely prepared recipient beds are useful for delivery and safety, but differ from chronic geographic atrophy, aged Bruch’s membrane and choriocapillaris loss.

These models also show why duration must be attached to an endpoint. Terminal graft survival can coexist with loss of an earlier ERG or behavioural advantage. Conversely, a negative whole-eye assay may miss local epithelial activity. The relevant record is therefore the duration of donor survival, topology, donor function and host benefit separately. Earlier rescue should not be erased by later failure, but neither should it be described as durable replacement.

### 4.4. Human Evidence and Its Limits

Early hESC-RPE suspension studies established delivery feasibility and reported pigmentation and visual changes in some eyes, but their small, open-label safety designs did not isolate efficacy [35,36]. The first autologous iPSC-RPE sheet recipient had persistent anatomy and stable acuity after neovascular-complex removal; a single surgically treated patient cannot establish a donor-mediated effect [37]. Two hESC-RPE patch recipients improved in acuity, reading speed and fixation after surgery for severe haemorrhagic neovascular age-related macular degeneration (AMD), with haemorrhage removal, vitrectomy, anti-VEGF treatment and residual retina remaining plausible contributors [38].

The bioengineered parylene programme provides the clearest example of why clinical anatomy and clinical efficacy must be graded separately. At one year, 15 participants had received an hESC-RPE implant in the worse-seeing eye. Implant localisation was stable, but mean acuity did not improve overall; five-letter responder comparisons with fellow eyes were not statistically significant, fixation was unchanged, microperimetry lacked a consistent true preoperative baseline, and serious ocular adverse events and early subretinal haemorrhage complicated interpretation [39]. At a median of three years, treated eyes were reported to gain more often and lose less often than fellow eyes, but the cohort remained single-arm, and the long-term clinical report could not establish living donor-cell survival or donor-specific physiology [40]. The same programme’s two-year postmortem specimen securely demonstrated donor persistence and interface activity in one eye, while vision remained count-fingers and unchanged [15]. Together, these reports establish complementary facts: the implant can harbour long-lived donor RPE, yet neither group-level visual benefit nor a donor-mediated mechanism has been demonstrated.

Later reports retain similar attributional constraints. Five-year patch follow-up showed a retained ten-letter gain in one recipient and decline below baseline in the other despite persistent imaging [29]. Three iPSC-RPE strip recipients had graft-associated anatomy without consistent microperimetric or clinically meaningful improvement; immune reaction, epiretinal membrane and oedema complicated interpretation [41]. In a six-participant retinal pigment epithelial stem cell-derived RPE (RPESC-RPE) cohort, the three worse-seeing eyes had larger reported gains than the three better-seeing eyes, but follow-up differed, microperimetry failed and no concurrent control or imaging marker authenticated the non-pigmented cells [42]. These observations justify further testing while leaving donor-mediated efficacy unresolved.

Safety and rare-disease series reinforce the importance of co-interventions. In one *RPE65*-associated Leber congenital amaurosis (LCA) recipient, cells migrated into an epiretinal membrane, pressure reached 46 mmHg and vision transiently fell to light perception; subsequent changes occurred in both eyes after several interventions [43]. A phase I Stargardt study reported non-significant acuity change with concurrent cataract surgery in some eyes [44]. Three-year follow-up in three Asian Stargardt recipients described one improved and two stable eyes using fellow-eye and external natural-history comparisons [45]. Such reports inform safety and feasibility without supplying a controlled efficacy estimate.

The nine-year postmortem study further illustrates the limits of metabolic inference. NAD(P)H fluorescence-lifetime measurements followed fixation and deparaffinisation, were not donor-cell resolved, and showed the same macula-versus-periphery direction in both eyes. Eight sampled regions per location were subsamples from one person, not independent biological replicates [16]. This evidence cannot establish donor-autonomous metabolism, although it documents unusual long-term graft-site anatomy. The two-year HLA-mismatched specimen provides stronger donor identity and interface evidence, but no direct transport or retinoid-cycle measurement and no visual gain [15].

Overall, the RPE literature supports product competence, selected examples of secure donor survival, and supportive epithelial-interface activity. Animal functional outcomes commonly reflect preservation of host retina; human evidence chiefly establishes feasibility, risks and anatomical persistence. No study in the reviewed corpus completes a demonstrated epithelial replacement mechanism together with convincing donor-mediated visual restoration. This is a limit of the available evidence, not a finding that the grafts are biologically inactive.

## 5. Photoreceptor Replacement

### 5.1. Provenance and the Transmitting Neuron

The required mechanism is donor light detection followed by transmission into the host retina. Outer-retinal placement and polarity should allow RPE support apically and access to bipolar or horizontal cells basally. Somatic migration into the host outer nuclear layer is not indispensable if a graft transmits across its boundary, and anatomically incorporated cells need not be physiologically connected [1,4,7,8].

Seminal precursor-transplantation studies established the biological plausibility of outer-retinal repair. Reporter-positive cells were found within the host outer nuclear layer with photoreceptor morphology, and later experiments combined apparent integration with light responses and improved visually guided behaviour [46,47]. These findings shaped the field, but their original cellular interpretation became insecure when two independent studies showed that donor and host photoreceptors can exchange cytoplasmic contents after transplantation [10,11]. Donor fluorophores, transcripts or proteins in a host cell can therefore produce an apparently integrated, reporter-positive photoreceptor without movement of the donor nucleus. The functional observations in the early studies are not erased retrospectively; rather, they demonstrate transplantation-associated rescue whose attribution to a new donor neuron is no longer secure. A 2024 author correction to the 2012 report addresses duplicated image content caused by montage misalignment; this image correction is distinct from the subsequent material-transfer reinterpretation [47].

The corrective lesson is also narrower than the claim that all apparent integration is material transfer. Donor cells derived from pluripotent stem cells can both integrate genuinely and exchange material, with the balance influenced by donor stage and recipient environment [12]. Human organoid-derived photoreceptors transferred cytoplasmic material to other human photoreceptors in vitro, whereas transfer to mouse retina was not detected in vivo [48]. This species asymmetry is reassuring for the interpretation of some xenografts, but it is not a general solution: a barrier that suppresses human-to-mouse exchange may disappear in a future human allograft. Material transfer must consequently be treated as a context-dependent competing mechanism, tested rather than assumed either present or absent [9,48].

Secure lineage must therefore accompany physiology. Cytoplasmic fluorophores, isolated donor proteins and opsins cannot alone authenticate the recorded cell. Orthogonal nuclear or genomic evidence, cell boundaries and continuity from the donor soma strengthen attribution. This is particularly consequential when only a few cells respond or when results are extrapolated from xenografts to human allografts.

### 5.2. Product Competence, Topology and Physiological Tests

Product assessment should quantify rod or cone identity, developmental stage, viability and contaminating or proliferative populations. In vitro light responses support M0; performance after transplantation requires separate evidence. Large-animal studies have securely identified human cells for up to 16 weeks in ground squirrels and 22 weeks in dogs, while exposing variable retention, ectopic localisation and dependence on immunosuppression [49,50]. Candidate pedicles and bipolar contacts were anatomical findings. An imaging-biomarker study likewise related structural grades to histology without testing donor or host physiology [51].

Synaptic-marker apposition specifies a candidate interface. Pikachurin puncta at graft–host contacts in late-stage rd1 retinas were not accompanied by ultrastructural or physiological transmission tests [52]. Electron microscopy can resolve specialised contacts, but function still requires a signal. For M2, donor-resolved recordings must identify intrinsic phototransduction. For M3-PR, an identified host response must be linked temporally and spatially to donor activity, with a control that discriminates transmission from coincident host function. Appropriate retinal pharmacology is useful, but residual host photoreceptors can use the same pathways. One experiment may inform M2, M3 and O4A only if source, transfer and host output are separately interpretable.

A phototransduction-deficient, viable donor comparator is particularly informative because it can retain surgery and cell exposure while disabling the proposed light sensor. Differences in survival, architecture or trophic action must still be measured. Reversible donor-selective silencing would strengthen necessity attribution but is an advanced test, not the only acceptable route to convergent evidence. Graft-centred physiology, secure identity, disease-stage controls and pathway perturbation can support a restricted coupling claim when direct donor switching is unavailable.

### 5.3. Strongest Local Physiological Evidence

Among the present corpus, transplantation of purified human pluripotent-stem-cell-derived cones into rd1/*Foxn1*-null mice provides one of the strongest donor-mediated packages. Human nuclear identity, nascent outer-segment morphology and graft-centred micro-electroretinographic responses were combined with light-responsive host ganglion-cell activity. Reversible glutamatergic blockade tested the relay, while phototransduction-deficient *CNGB3* donor cells acted as a mechanistically relevant control [17]. These contrasts make a purely paracrine explanation inadequate and support M3-PR and local O4A with high causal confidence. The residual uncertainty is narrower: donor cells were not switched off directly, the xenograft model does not reproduce human immune or synaptic conditions, and many recorded units were nested within five to six retinas.

A related study in end-stage *Aipl1*-null mice again found graft-centred micro-electroretinographic signals and fast host ganglion-cell responses that were reversibly abolished by retinal synaptic blockers [18]. All four technically successful treated retinas showed local physiology, whereas full-field ERG was negative. Optomotor thresholds were measurable in eight of 15 treated eyes and were not reproduced at every session. This pattern is biologically coherent for a small focal graft: local M3-PR/O4A can be persuasive, while a global retinal assay lacks sensitivity and behaviour remains variable. It also prevents overstatement. Four successful retinas do not establish a general response rate, and the behavioural result merits lower causal confidence than the spatially resolved physiology.

Human iPSC-derived cones transplanted into the Cpfl1 cone-degeneration model provide convergent but less source-specific evidence. Human nuclear and mitochondrial markers supported donor identity, transplanted cones polarised and developed segment-like structures, and photopic host retinal responses were reported in five of nine grafted eyes with sensitivity to ON-pathway blockade [53]. However, the recipient retains rods, so host light detection and rod–cone pathway interactions remain more plausible alternatives than in an end-stage pan-photoreceptor model. The study supports functional coupling at moderate causal confidence; it does not by itself prove that every recorded response originated in a donor cone.

The same local-versus-global pattern has now been reproduced in 12- to 15-month-old rd1 mice, a more stringent test of age and advanced remodelling. Approximately three months after human cone transplantation, all four technically successfully treated retinas showed graft-local responses on multielectrode recording with reversible synaptic blockade, while eye-specific optomotor thresholds were measurable in five of nine treated eyes [54]. The experiment strengthens model relevance and durability relative to young recipients, but remains a small xenograft study without donor-selective silencing; recording assessment was not fully masked and paired eyes were not analysed with a paired model. Its defensible contribution is replication of focal M3-PR/O4A under a more hostile recipient condition, not a claim of complete or uniform visual restoration.

Together, these studies establish that selected human cone grafts can recruit host retinal pathways in severely degenerated mice [17,18,53,54]. Their strongest evidence concerns focal physiology; the probability of success per treated eye, response durability and behavioural generalisability are less certain. A negative full-field ERG in this setting is not evidence against the demonstrated focal response. Conversely, recording several successful preparations does not estimate efficacy across every attempted transplant.

### 5.4. Rescue with Unresolved Cellular Attribution

Other positive studies expose different inferential gaps. One precursor-transplantation study classified 15 of 30 animals as behavioural responders and concentrated subsequent assays in that subset [55]. This supports observations in selected responders, not an unselected treatment response rate. An unsorted early-organoid cell product produced pupillary differences at some irradiances in rd10 mice, but relied on a cytoplasmic donor reporter, anatomical host proximity and an unpresented full-field ERG comparison [56]. Residual photoreceptors, intrinsically photosensitive RGCs and trophic or transfer effects remained plausible.

Combined RPE–photoreceptor products require component-level interpretation. In sodium-iodate injury, only hESC-RPE and mouse photoreceptors together in hydrogel met the ERG response criterion and improved scotopic behaviour; useful component controls identified an effect of the combination, but donor rods were sparse and immature and physiology was not donor resolved [57]. A mixed hiPSC-derived product administered before expected degeneration in *Pde6b*-knockout rats preserved ERG responses for up to nine months without demonstrating component-specific function or donor-to-host transmission [58]. Preservation was established more clearly than reconstruction of an absent circuit.

Survival mechanisms also have restricted scope. Donor and host *Ripk3* perturbations, including bone marrow chimeras, altered four-week precursor survival, while host photoreceptor protection after retinal detachment occurred without transplantation [59]. These experiments inform cell-death and immune biology without demonstrating phototransduction or integration. Study details and assay limitations are retained in the Supplementary Material.

### 5.5. Disease Stage and the Claim Supported

Early disease preserves postsynaptic partners but makes host-cell rescue a stronger alternative. End-stage degeneration reduces residual photoreceptor ambiguity while increasing glial sealing, dendritic retraction and retinal remodelling. The intended intervention should therefore be described as preservation, supplementation or replacement after cell loss. Larger eyes improve surgical relevance but require their own physiological validation [49,50].

For a photoreceptor-replacement claim, the minimum evidential package is secure donor identity and suitable topology, evidence attributable to donor light detection, and physiological recruitment of identified host retina with a discriminating transmission control. The strength of attribution depends on how well the design excludes residual host detection, material transfer and trophic preservation. Donor-specific loss of function offers stronger discrimination where feasible; it should not be confused with a universal requirement for every informative experiment. Anatomical contacts alone remain supportive, and an unmeasured interface remains unassessed.

Outcome testing should match the donor phenotype and graft field: rod-mediated dark-adapted function for rod products and cone-mediated photopic function for cone products, with stable fixation, repeated testing and prespecified loci. Visual acuity alone cannot identify the source of a small functional island. No completed human study in the reviewed corpus demonstrates clinically meaningful benefit attributable to an isolated photoreceptor-cell graft. The justified conclusion is convincing local preclinical coupling in selected models, with treatment-level reliability and human efficacy still to be established.

## 6. Retinal Sheets and Organoids

### 6.1. Defining the Functional Unit

Retinal sheets preserve developing tissue relationships but contain several potentially active populations, including photoreceptors, interneurons, glia and progenitors. A product can mature internally, preserve host neurons and transmit signals through different routes. Its evaluation therefore needs three explicit resolutions: the whole graft, each donor cell class invoked by the claim, and the relevant donor–host interface [1,8]. These are alternative levels of description, not additional ranks in the framework.

For photoreceptor replacement, the intended route is donor photoreceptor to host bipolar or horizontal cell, followed by host retinal output. If donor photoreceptors first drive donor bipolar cells, a further output across the donor–host boundary must be demonstrated. Internal donor–donor synapses establish graft maturation, not host coupling. A donor-network light response can be reported as such without assigning donor-photoreceptor M2 when the recorded cell is unresolved. Similarly, M3-composite denotes supported physiological coupling at product level with unresolved cellular contributors; it must not be inferred from behaviour or global electrophysiology alone.

The minimum composite-graft claim should therefore specify product composition, authenticated donor tissue, the source of the measured activity and evidence that it crosses into host circuitry. Attribution to photoreceptors or another named population additionally requires evidence resolving that population’s contribution. Demonstrating every internal graft synapse is unnecessary for a restricted product-level claim, but broader retinal reconstruction requires evidence for the additional interfaces it invokes. This approach accommodates useful composite function without mislabelling it as replacement by a single cell class.

### 6.2. Composition and Topology

Manufacturing studies show why tissue identity cannot be assumed from external appearance. Retinal differentiation can also yield eye-related, cortex-like and spinal-cord-like tissue. Testing a peripheral ring while retaining the central cap for transplantation offers a practical quality-control strategy, but relies on the sampled region representing the implanted tissue [60]. Single-cell analysis after transplantation identified donor astrocytes and brain/spinal-cord-like precursors that migrated through host retinas, whereas donor photoreceptors remained predominantly subretinal [61]. The study did not distinguish selection of pre-existing cells from host-induced change. Both findings support continued composition surveillance after release testing.

Lamination is similarly informative but not an ordinal proxy for function. In a 2026 comparison, both initially non-layered and well-layered *RAX*-reporter spheroids developed photoreceptor layers after transplantation into S334ter rats, and host ganglion-cell light responses occurred in two of three retinas in each formulation at approximately 300–330 days [62]. The result suggests substantial host control over graft organisation. It does not establish equivalence between formulations: three eyes per group, two responders in each, no formal equivalence margin and *RAX*-positive off-target potential are insufficient to conclude that initial lamination is irrelevant. Topology should be tested as a prespecified predictor of a functional route, not used as a stand-alone evidence level.

Rosettes can contain locally polarised photoreceptors while separating outer segments from host RPE and presenting irregular synaptic surfaces to host bipolar dendrites. Correctly oriented area, interface length, lesion coverage, RPE contact and ectopic growth are consequently more informative than a binary label of “laminated”. However, topology is a predictor to be tested, not a surrogate for coupling. Secure provenance must accompany the participating cells, including both sides of any claimed donor–host synapse; tissue format does not remove the material-transfer problem.

### 6.3. Physiological and Component-Specific Evidence

The early proof-of-concept mouse study by Mandai and colleagues placed iPSC-derived retinal sheets into end-stage rd1 retinas and combined graft maturation, genetically labelled candidate synapses, graft-local electrophysiology, host ganglion-cell responses and visually guided behaviour [63]. L-AP4 sensitivity supported recruitment of an ON-bipolar pathway, and unsuccessful grafts and untreated rd1 mice provided useful comparators. The study nevertheless selected structurally successful grafts by optical coherence tomography for parts of the functional analysis, used behavioural training and 9-cis-retinol without a matched grafted washout contrast, and did not silence the donor. It supports M3-PR/composite, O4A and O5A at moderate confidence; it does not show that donor photoreceptors were necessary for every recovered response.

Engineering graft composition is a more discriminating test than comparing transplantation with no transplantation. Mouse embryonic stem cell (ESC)/iPSC sheets with reduced secondary retinal neurons retained a photoreceptor layer, increased host-graft contact, altered host ganglion-cell responsiveness and reduced aberrant spiking relative to wild-type grafts [64]. The multielectrode analysis appropriately used a mouse-level hierarchical model despite 5738 recorded cells from 38 mice. However, deletion of *Bhlhe23* (formerly *Bhlhb4*) or *Isl1* changes a tissue rather than switching off one synapse: architecture, spontaneous activity, cell competition, trophic output and host preservation may all change. An internal inconsistency between the reported behavioural total and the listed subgroup totals further limits the precision of the O5 estimate.

The human-cell extension of this strategy used *ISL1*-null hESC-derived sheets with fewer graft ON-bipolar cells in end-stage S334ter rats [19]. Across 47 retinas, response probability followed the order non-transplanted control, wild-type graft and *ISL1*-null graft; host ganglion-cell responses were L-AP4 sensitive and recordings were centred over the graft. Human nuclear markers and the engineered composition secured the donor context. Yet *ISL1* deletion also improved host–graft contact and preservation of host inner plexiform structure, and direct donor contribution to host output remained unresolved. This is one of the field’s strongest causal interventions, but its defensible conclusion is that graft composition affects local coupling, not that the intended donor-photoreceptor-to-host-bipolar connection is the unique mediator.

Manufacturing and mechanistic evidence should also remain separate. The quality-controlled iPSC-sheet programme combined product assays, human nuclear provenance, long-term safety and graft-centred multielectrode recordings in 12 transplanted and 12 control retinas [60]. Robust responses were present in only two of 12 grafted retinas before L-AP4 and in six of 12 after washout. The post-washout prevalence is informative about latent pathway capacity but should not be presented as the untreated functional response rate. This study provides strong M0 evidence and moderate M3-composite/O4A evidence while illustrating why baseline, pharmacologically suppressed and experimentally unmasked states require separate reporting.

These comparisons are more informative than transplantation-versus-no-transplantation alone because they alter a plausible contributor to coupling. They do not isolate one synapse when the same change affects graft composition, architecture, spontaneous activity and host preservation. The causal conclusion should therefore name the manipulated component and the measured consequence. It should not assume that all product-level benefit travels through one donor-photoreceptor-to-host-bipolar route.

Correlative array tomography linked origin-resolved candidate synaptic structures to local host-RGC light responsiveness in rd1 mice [65]. The convergence supports M3-PR at moderate confidence, but ultrastructure involved two animals and the physiology–imaging cohort comprised 12; response classification and electrode-level nesting limited inference. One wild-type graft had candidate contacts without responsive host RGCs. Thus, the study strengthened the anatomical–physiological association without showing necessity.

Genome-edited sheets in six rd1 retinas produced light adaptation and some flicker following, extending O4A beyond detection of a flash response [66]. Responses remained slower or incomplete, few cells followed frequencies above 5 Hz, and two retinas had low responses attributed to technical problems. These findings describe recovered processing without resolving donor-photoreceptor M2 or the unique transmitting interface.

Horizontal cells further demonstrate that the active unit is a reconstructed microcircuit. Using horizontal-cell-depleted rd1 hosts and *Isl1*-null organoids that retained graft horizontal cells but lacked rod bipolar cells, serial electron microscopy identified triads containing graft horizontal-cell processes and host bipolar dendrites at graft photoreceptor ribbons [67]. Removing host horizontal cells did not reduce the counted host-bipolar-to-graft-photoreceptor contacts, but it reduced restored light sensitivity. This supports a strong restricted claim: graft horizontal cells can participate in synapse assembly, while host horizontal cells modulate the physiological result. It does not show that horizontal cells are sufficient for the overall response, because donor-specific silencing or ablation was not performed and hundreds of recorded ganglion cells were nested within 16 retinas.

The horizontal-cell experiment illustrates why a restricted contribution can receive strong causal support while overall replacement remains less securely attributed. Donor and host interneurons, photoreceptors and preserved host tissue may all participate. The framework should retain those distinctions rather than assign one grade to the entire product or paper.

### 6.4. Downstream Rescue and Model Dependence

Embryonic rat retinal sheets provided early evidence of spatially matched superior-colliculus responses and candidate ultrastructural contacts in S334ter degeneration, but selected animals, incomplete same-animal mapping and mixed cell content limited cell-specific attribution [68]. Human ESC organoid sheets subsequently persisted for 54–300 days with optokinetic and superior-colliculus responses in immunodeficient rats; masked scoring and sham controls strengthened the treatment association, while functional source attribution remained incomplete [69].

In RCS rats, graft-associated ERG benefit disappeared by four months, host cones were preserved near grafts, and superior-colliculus responses occurred in seven of 14 tested animals [70]. The primary host RPE defect makes preservation a particularly plausible mechanism. A shipping study transplanted 56 rats but analysed optokinetic outcomes in 14 and superior-colliculus responses in five, of which four responded [71]. Those findings inform transport feasibility and selected-graft activity, not the probability of functional replacement across the full surgical cohort. Additional denominators and selection details are retained in Supplementary Section S4.

These studies should not be dismissed because mechanism is unresolved. They establish tissue survival, treatment-associated responses or logistical feasibility. The narrower conclusion is that the available observations cannot determine which donor cells, host cells or non-replacement effects generated the response. Behavioural improvement remains a separate outcome rather than evidence completing an unmeasured host–graft interface.

### 6.5. Large-Animal and Human Evidence

In laser-injured macaques, major histocompatibility complex (MHC)-homozygous monkey iPSC sheets survived for three to six months, but peripheral assays suggested subclinical rejection in three of four mismatched animals, and restored primate physiology was not demonstrated [72]. Functional findings from a separate mouse arm cannot establish coupling in the primates. In cobalt-chloride-injured minipigs, correctly oriented tissue in one eye coincided with local multifocal-ERG improvement at days 45 and 60, whereas a folded graft did not [73]. The spatial contrast was informative, but one positive eye, immature outer segments and an acute lesion constrained attribution and generalisability.

A single-macaque macular-hole graft closed an experimental defect while fixation and focal electrophysiology changed over approximately 200 days [74]. No host–graft synaptic connection was confirmed, the untreated fellow lesion also improved, and mechanical closure was a plausible explanation. Anatomical repair therefore should not be equated with neuronal replacement.

The strongest current large-animal evidence comes from *ISL1*-null human organoids transplanted into an acute laser-ablation model of the macaque macula [75]. Host rod- and cone-bipolar dendrites extended towards graft photoreceptors, and immunohistochemistry and ultrastructure supported cross-origin contacts. Focal b-wave responses improved by up to 21.6% in two of four engineered-graft eyes and remained stable for up to two years; a delayed d-wave increase occurred in one of those eyes. This reaches M3-PR and O4A with moderate causal confidence and important durability. It does not reach donor-resolved M2 or visual restoration: the a-wave did not show a corresponding change, the lesion was acute, the cohort was very small, one engineered-graft eye developed endophthalmitis, a wild-type graft was partly lost, protocols changed during follow-up and no donor-off test was performed.

The primate findings support durable focal physiology in a subset, without establishing perceptual use or a treatment effect in chronic macular degeneration. Their translational value concerns surgical scale and persistence of a local response; lesion chronicity, the treated-eye denominator and meaningful visual endpoints remain separate questions.

The first human allogeneic iPSC-sheet study involved two participants with advanced retinitis pigmentosa. Graft-associated structure persisted for two years without a serious graft-related adverse event, while apparently slower decline than in fellow eyes remained exploratory [76]. Non-randomised treatment, inter-eye asymmetry and residual function prevented a controlled efficacy inference. Adaptive-optics OCT later resolved rosette-like features and an outer-plexiform-layer-like line in one participant at approximately two years three months [77]. Imaging can monitor candidate structures but cannot authenticate donor nuclei or demonstrate transmission; resemblance to histology from another animal cannot supply that evidence in the patient.

Selected rodent studies therefore support local M3-PR or composite coupling and O4A, with the engineered-graft primate study adding durable focal evidence in two of four eyes [19,60,63,64,65,66,67,75]. Human evidence remains principally anatomical and safety-oriented. Where donor cell classes or transmission routes are unresolved, the appropriate conclusion is unresolved attribution at that resolution, not absence of graft activity. Future testing should isolate the proposed source or relay while measuring any accompanying changes in survival, tissue structure and host preservation.

## 7. Retinal Ganglion Cell Replacement

### 7.1. Retinal Input and Central Output

RGC replacement requires both retinal afferent input and functional output to central visual targets. Donor dendrites must receive appropriate bipolar and amacrine signals, while axons must enter the optic nerve, navigate the chiasm, reach appropriate targets and form transmitting synapses. The framework treats these as separate M3 modules because their anatomical distances, experimental tests and current evidence differ substantially [5,20,21].

M2 for a conventional RGC concerns post-transplant membrane properties, excitability and action-potential generation. A synaptically driven light response instead supports M3-RGC-A. An explicitly specified intrinsically photosensitive RGC product would require separation of intrinsic and circuit-driven light responses. Similarly, O4B records downstream pathway activity without authenticating its donor source: preserved host axons could carry the signal. Complete conventional-RGC replacement requires both interfaces, while a restricted afferent-integration claim need not imply central reconstruction.

### 7.2. Product Identity, Access and Anatomical Milestones

Manufactured RGC-like products express combinations of BRN3B, RBPMS, SNCG, THY1 and neuronal markers; several show excitability or spontaneous synaptic currents in vitro [78,79,80,81,82]. These support product competence without establishing adult subtype identity or target-specific projection capacity. Directly induced human cells, for example, resembled fetal rather than adult RGCs transcriptomically [83]. The intended subtype and visual role should therefore be stated where known. In vitro neurite growth or firing cannot by itself predict optic-nerve entry, sustained conduction or target selection.

The internal limiting membrane (ILM) and Müller endfeet restrict entry after intravitreal delivery. Enzymatic or mechanical disruption increased donor access to the ganglion-cell and inner-plexiform layers [84]. This is a barrier mechanism, not physiological integration. Low-dose plasmin increased donor detection at one week but not one month, while higher doses impaired host electrophysiology [85]. Laser photodisruption and collagenase improved some short-term access measures in bovine and human explants while also injuring host cells or permitting ectopic penetration [86]. Aguzzi and colleagues extended the barrier observations across rodent, macaque and human-explant preparations [20]. Each strategy requires matched interface-disruption controls because host injury can alter the same physiology used to assess integration.

Early transplantation studies established sparse, often short-lived donor residence and processes, with limited survival or neurite persistence and no donor-resolved afferent physiology [78,79,81,82]. Longer observation did not necessarily improve the functional evidence. Mouse pluripotent stem cell (PSC)-derived RGCs remained detectable at 12 months in two of four long-term recipients, with overall survival at most approximately 5%, cellular overgrowth and frequently short or disorganised processes [87]. A biodegradable scaffold supported human retinal neurons and optic-nerve-directed projections in primates for up to a year without demonstrating functional central reconnection [88]. These results concern persistence, delivery and anatomical route access.

Developing donor RGCs and ESC-derived retinal fragments have also shown dendritic entry and candidate contacts, including presumptive synapses in selected N-methyl-D-aspartate (NMDA)-injured retinas [89,90]. Such anatomy identifies where afferent physiology might be tested; it does not establish synaptic strength, receptive-field organisation or light-driven spike output. Reporting should distinguish recipients attempted, surviving donor cells, correctly localised cells and successfully recorded cells. Sparse-cell proof of principle is informative, but does not measure integration efficiency across a whole product.

### 7.3. Provenance and Afferent Physiology

After ILM disruption, human RGC xenografts transferred cytoplasmic, nuclear-associated and mitochondrial labels to host Müller glia [91]. The events were rare, yet potentially consequential when functional claims rest on a few labelled cells. The finding does not invalidate every donor recording. It requires orthogonal identity evidence in the recorded soma, with continuity used to assign processes and axons, rather than reliance on an isolated transferable marker.

Direct recording from an identified transplanted RGC is the most informative assay of M3-RGC-A because a conventional RGC cannot become light responsive without input from the retinal circuit. In the 2016 primary-cell study, five donor cells from three retinas showed weak, adapting ON, OFF or ON-OFF responses [92]. The recordings established proof of principle that an adult recipient retina can supply functional afferent input to a transplanted neuron. The very small recorded sample, healthy host circuitry and use of developing primary cells limit generalisation to a manufactured human product or a chronically glaucomatous retina.

Human iPSC-derived RGCs subsequently provided a second donor-resolved signal. At five months, mean reported survival was 0.134%, nine of 16 mice met a threshold of at least 0.1% survival, and four of six recorded donor cells from four retinas were light responsive [93]. This supports M3-RGC-A with moderate confidence in the surviving, recordable subset. Four cells are not evidence that the product integrates efficiently, and selection for cells accessible to two-photon imaging and recording conditions the response fraction on prior survival and visibility. The study did not test optic-nerve reconstruction or behaviour.

A 2026 study provides the strongest current afferent package. Across 28 ILM-defective retinas, 73 light responses were detected among 2553 donor cells, compared with one among 247 donor cells in ten intact-ILM retinas; cobalt chloride blocked the responses [20]. Secure human donor context, dendritic entry into the inner plexiform layer (IPL), the intact-ILM comparator and synaptic blockade jointly support an A-grade restricted claim that ILM disruption permits functional host input to a subset of donor RGCs. The counts also prevent exaggeration: the responding fraction was approximately 3% in disrupted preparations, responses were calcium signals rather than direct spike-output recordings, and multiple cells were nested within each retina. This is persuasive M3-RGC-A, not population-wide integration.

These experiments support host input to selected transplanted neurons. Their remaining limitations concern efficiency, response quality, durability and model applicability, rather than an absence of all afferent integration. Generic ON or OFF modulation does not establish adult subtype-appropriate receptive fields or naturalistic visual coding, and synaptic suppression may not identify the immediate presynaptic cell. Direct donor voltage or spike recordings, receptive-field mapping and co-registered dendritic anatomy would strengthen functional characterisation without changing the restricted conclusion already supported.

For an M3-RGC-A claim, the minimum evidence is an authenticated donor cell with access to the appropriate retinal input layers and a physiological response attributable to host synaptic transmission. Intrinsic photosensitivity and reporter transfer must be excluded where relevant. Detailed subtype mapping and longitudinal recordings strengthen the claim but should be distinguished from the initial demonstration that host input is possible. The fraction of responsive cells must be reported within the fractions that survived and were technically recordable.

### 7.4. Efferent Reconstruction

No study in the corpus satisfies M3-RGC-B. Preliminary donor-labelled axons were described in the optic tract, lateral geniculate nucleus and superior colliculus after primary RGC transplantation [92], while other studies showed short retinal processes, growth towards or into the optic-nerve head, or projections along the nerve-fibre layer [20,82,87,88]. These observations demonstrate growth potential and route access. They do not establish that a continuous donor axon traversed the full pathway, selected an appropriate target, formed a transmitting synapse or preserved retinal topography. Optic-nerve-head entry is the beginning of efferent reconstruction, not its completion.

The relevant test is whether an authenticated donor axon conducts to an appropriate central target and forms a functional output synapse. Continuous lineage tracing, target-specific terminals and donor-evoked postsynaptic activity would jointly support this claim; eye-evoked cortical or superior-colliculus responses cannot identify donor output when host axons remain. Donor-selective activation can test sufficiency, while reversible output silencing can test necessity. Retinotopic correspondence should be measured for claims about appropriately organised visual output.

These experiments face a different feasibility problem from retinal recordings: long axonal distances, an inhibitory adult optic nerve, route selection and potentially altered central targets. Short retinal processes, optic-nerve-head entry and isolated central reporter-positive profiles remain anatomical milestones. Failure to demonstrate the complete route is an evidential gap, not proof that every donor axon fails. Nevertheless, no study in the reviewed corpus supplies the combined physiological evidence required for M3-RGC-B, donor-attributed downstream activity and useful visual behaviour.

### 7.5. Neuroprotection and Disease Relevance

RGC transplantation has a particularly credible non-replacement route because donor cells can protect host neurons whose axons already reach the brain. Directly induced fetal-like human RGCs preserved approximately 200 additional host RGCs per square millimetre after optic-nerve crush despite fewer than 100 surviving donor cells per retina [83]. The numerical mismatch, acute injury context and absence of donor-pathway reconstruction support neuroprotection rather than one-for-one replacement. This is a biologically meaningful treatment effect. Labelling it RGC replacement would conceal the mechanism most consistent with the data.

Host RGC survival, optic-nerve axons and host-specific physiology should therefore accompany donor measurements. Secreted factors, immune changes and interface manipulation can preserve or alter the existing pathway. Conditioned-medium, non-functional-donor and procedure controls can narrow these alternatives, but differ in trophic exposure and inflammatory effects. A matched, reversible donor-output perturbation would provide stronger discrimination if it leaves graft health and host reserve otherwise comparable.

Healthy retina supplies intact presynaptic partners and tests access to a functioning circuit. Acute NMDA injury and optic-nerve crush address selected mechanisms but do not reproduce chronic glaucoma or inherited optic neuropathy. A staged programme should distinguish retinal access, afferent competence, axonal navigation and central connectivity, using disease models appropriate to each question. Large-animal work adds surgical scale and distance without automatically reproducing chronic disease. The practical requirements for a complete pathway claim exceed those for an informative early mechanistic study.

There is no human efficacy evidence for stem-cell-derived RGC replacement in the reviewed corpus. Early clinical work would address delivery and safety, while visual-field or electrophysiological change would remain exploratory without an appropriate comparator and mechanistic support. The evidence presently supports rare-cell afferent coupling and anatomical growth milestones; it does not establish reconstruction of a usable donor retinofugal pathway. This conclusion preserves the importance of the afferent advance while keeping the unresolved output interface explicit.

## 8. Alternative Explanations and Assay Interpretation

### 8.1. Competing and Coexisting Mechanisms

**Figure 3:**
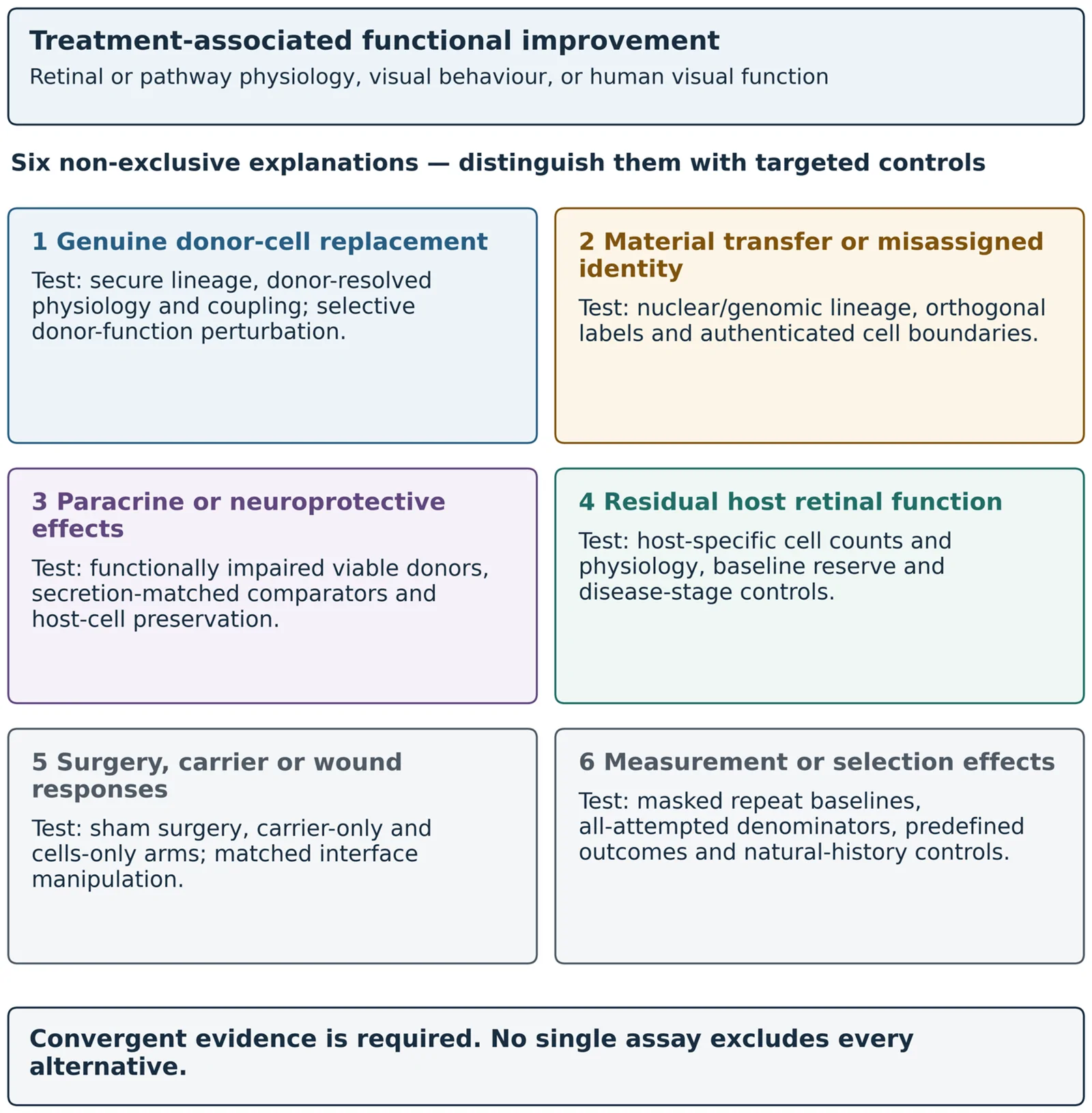
Non-exclusive explanations for treatment-associated improvement and examples of discriminating tests. These tests should be selected according to the product and claim; donor-selective perturbation is a stronger mechanistic test where feasible, not a universal clinical requirement. Alternative mechanisms can coexist with replacement.

The observed treatment effect can arise from living donor function, transferred material, trophic or immune action, host preservation, surgery, carrier effects, concomitant treatment and measurement variability. These mechanisms may interact and may coexist with genuine replacement. Their plausibility depends on the product, disease stage and measured endpoint (Figure 3). The question is which alternatives the study can distinguish, rather than whether every conceivable confounder has been eliminated.

Material transfer primarily threatens the bridge between a labelled cell and its assigned donor identity [9,10,11,12,91]. It does not automatically invalidate a functional outcome. Paracrine effects instead threaten attribution of host recovery to the proposed replacement function. A viable graft can preserve host photoreceptors or RGCs without performing the missing cell’s physiological task. For RPE, host photoreceptor preservation may also be the expected consequence of authentic epithelial replacement, so the discriminating evidence must concern donor epithelial activity. For neuronal grafts, baseline and terminal host-cell measurements help identify whether an apparently restored signal could originate in surviving host neurons.

Residual reserve and model stage must be interpreted together. Early intervention offers viable partners but stronger preservation alternatives. End-stage disease reduces residual-cell ambiguity while increasing remodelling and loss of receptive circuitry. Neither is universally the better model. The study should specify which host cells were absent, which remained, and whether the intention was preservation, supplementation or replacement. Heterogeneous degeneration limits the ability of histology from separate animals to establish the substrate in each functionally tested eye.

Surgical and carrier effects need equally specific controls. Subretinal blebs, debridement, haemorrhage removal, vitrectomy, cataract surgery, repeated anti-VEGF treatment and scaffolds can alter anatomy or vision independently of the proposed donor function [25,26,27,28,29,30,38,39,40,41,42,43]. ILM disruption may facilitate RGC entry while damaging the host substrate [20,85,86]. Immunological interventions can improve survival without demonstrating physiological integration [13,33,34,72]. Controls should target the active components of the actual intervention rather than assume that the delivery procedure is neutral.

### 8.2. What Assays Can Establish

An assay’s evidential role depends on identity, stimulus and spatial resolution. Direct intrinsic donor physiology can support M2; a donor RGC response dependent on host input can support M3-RGC-A; and a whole-retinal response can support O4A while leaving its source unresolved. Electron microscopy supplies anatomical evidence, whereas transmission requires physiology. Focal electrophysiology improves concordance with a small graft but does not by itself exclude protected host cells. Table 3 provides a compact guide; the detailed assay and safeguard matrix is retained in Supplementary Table S1.

Downstream recordings and behaviour add biological relevance but usually lose cellular specificity. A cortical response demonstrates that a signal reached the brain; an optomotor response demonstrates use of available visual information. Neither identifies the donor cell or its interface without additional evidence. Conversely, a negative global or behavioural result may reflect limited graft area, an insensitive task or a damaged downstream pathway despite real local coupling. Positive and negative findings should therefore be interpreted against what the assay could detect under the conditions tested.

### 8.3. Spatial, Temporal and Statistical Interpretation

Spatial agreement between graft and response tests a prediction of local replacement, but trophic and procedural effects can share the same location. Graft boundaries, residual host retina, recording sites and functional loci should be registered prospectively, with non-graft comparisons retained. Selecting only the region with the strongest post-treatment response converts the analysis into an exploratory assessment.

Durability likewise concerns several trajectories: donor survival, correct topology, intrinsic function, host coupling and visual benefit may peak or fail at different times. A late anatomical specimen cannot authenticate earlier physiology, and an early peak cannot establish terminal function. Reports should distinguish the last observed graft from the last demonstrated functional effect and account for attrition.

The recipient is usually the independent treatment unit. Cells, image regions, electrodes and repeated trials increase information within a recipient without creating additional independent grafts. Mixed-effects models, hierarchical resampling or recipient summaries can accommodate nesting. Randomisation, masking, prespecified exclusions and complete denominators remain necessary for interpreting response frequencies. Analyses restricted to technically successful or behaviourally responsive grafts can answer mechanistic questions, but should not be presented as population-level treatment success.

### 8.4. Testing Donor Participation

A useful sequence is to authenticate the donor, document residual host capacity, register the graft and response, test the required interface, and challenge the leading alternative mechanism. Donor-selective activation or reversible silencing can then strengthen sufficiency or necessity claims where feasible. No single perturbation is intrinsically decisive: ablation may inflame the retina, silencing may alter secretion, and genetic changes may alter graft composition or survival. Comparable graft amount, health, location and host reserve should be demonstrated rather than assumed.

If a properly targeted perturbation abolishes and restores the predicted local response, attribution becomes stronger. If the response persists, the targeted function may not be necessary under those conditions; this does not automatically exclude a smaller or redundant donor contribution. If target engagement, assay sensitivity or graft viability is uncertain, the result remains inconclusive. This distinction between absent evidence and discriminating negative evidence should govern both the narrative assessment and the framework labels.

## 9. From Preclinical Rescue to Human Efficacy

### 9.1. Matching the Model and Clinical Claim

Translation can fail because the tested mechanism differs from the clinical need. A young rodent preservation model offers a receptive substrate and residual photoreceptors, whereas chronic geographic atrophy includes aged matrix, choriocapillaris change and regions with absent photoreceptors. A small cone graft can recruit mouse bipolar circuits without covering a useful human macular field. An RGC receiving local input still faces a much longer diseased optic pathway in humans. Species, lesion chronicity, residual substrate, immune context and surgical scale should therefore be considered separately.

No single model answers every question. Healthy or acute-injury tissue can test delivery and interface access; chronic degeneration can test remodelling; large eyes can test device geometry and anatomical distance. Convergent results across appropriate models are more informative than assigning one species a universally higher evidence status. The intended clinical claim should determine which untested links matter most.

Clinical endpoint selection should follow the proposed function. For RPE, sensitivity over the implanted field is a plausible consequence in surviving host photoreceptors but is not donor epithelial physiology. For photoreceptors, local retinal responses should map to the donor field and be linked preclinically to phototransduction and transmission. For RGCs, a pathway or field change requires support for both input and output interfaces. Imaging establishes placement and structural safety, local testing measures retinal consequences, and patient-centred outcomes establish whether the change matters; no one endpoint substitutes for all three.

### 9.2. Comparators and Clinically Meaningful Outcomes

Human studies in the reviewed corpus principally address safety and feasibility. Open-label, small single-arm or dose-escalation designs can reveal surgical complications, inflammation, migration and structural persistence but do not reliably isolate efficacy. Treatment of the worse-seeing eye, unstable baseline testing and concurrent cataract surgery, haemorrhage removal, anti-VEGF treatment or reoperation complicate visual interpretation [29,35,36,37,38,39,40,41,42,43,44,45]. A favourable course therefore remains an association unless supported by a credible comparison.

The fellow eye is informative but not automatically exchangeable: disease severity and fixation may differ, learning can affect both eyes and systemic immunosuppression is shared. External natural-history cohorts provide context with their own selection and measurement differences. When sham surgery is not acceptable, randomised delayed treatment, suitable device or dose comparisons, prospectively matched untreated cohorts or prespecified grafted-versus-non-grafted loci may provide partial counterfactuals. Their remaining limitations should be explicit.

Repeated preoperative measurements are particularly important in profound visual loss. Fixation, repeatability and floor effects determine whether a change can be interpreted. A postoperative recovery visit should not be substituted silently for the true preoperative baseline. Reports should retain all treated participants and distinguish treatment-assignment outcomes from outcomes conditional on a successful graft. Clinically meaningful thresholds, follow-up intervals, missing-data handling and the analysis population should be specified before examining results.

### 9.3. The Graft Field, Durability and Safety

Focal treatment calls for spatially registered assessment. Graft boundaries, atrophy, surviving retina, surgical injury and sensitivity loci should be mapped together across visits. Prespecified exposed loci and appropriate comparison loci can reduce dilution by untreated retina, although a focal trophic or procedural effect remains possible. A small sensitivity island may validate local biology without improving reading, navigation or stable fixation. Mechanistic success and patient benefit therefore require separate reporting.

Local tests also have limitations. Microperimetry depends on fixation and learning; full-field stimulus testing measures very low vision without localising its source; ERG, pattern ERG, photopic negative response and visual-evoked potentials interrogate different pathway components. OCT, fundus autofluorescence and adaptive-optics OCT cannot authenticate living donor cells from reflectance alone [16,77]. Clinical concordance can strengthen a mechanistic interpretation but cannot supply donor identity or physiological coupling that was never measured.

Safety and function should be followed on the same timeline. Long-lived implant visibility is distinct from durable donor physiology. Migration, membranes, multilayer growth, haemorrhage, oedema, retinal detachment, pressure elevation and rejection may disrupt the required topology or affect testing. Conversely, an adverse surgical course can obscure donor competence. Negative outcomes should identify whether the relevant function was tested adequately or rendered unassessable by loss of the host substrate.

### 9.4. Current Clinical Interpretation

RPE studies establish delivery feasibility, procedure-specific risks, persistent graft-associated anatomy and, in selected postmortem tissue, secure donor survival [15,16,29,35,36,37,38,39,40,41,42,43,44,45]. Retinal-sheet studies provide preliminary safety and anatomical evidence from two human recipients, with subsequent high-resolution imaging in one of these recipients [76,77]. Neither body of literature presently supplies convincing donor-mediated visual restoration, and the corpus contains no established human efficacy for isolated photoreceptor or RGC replacement. These conclusions concern the strength of demonstrated attribution; they do not establish that no recipient benefited biologically.

Mechanistic uncertainty and therapeutic efficacy should remain separate. A controlled clinical benefit can be meaningful even when donor participation is inferred partly from preclinical evidence. Conversely, elegant preclinical donor-necessity experiments cannot compensate for an uncontrolled human efficacy comparison. Direct donor silencing or tissue sampling may be inappropriate in living participants, so the feasible clinical package combines a credible counterfactual, spatially matched outcomes, stable baseline testing, safety surveillance and a relevant preclinical mechanism. Postmortem donation can add rare lineage evidence, but should not be a prerequisite for recognising benefit.

## 10. Practical Requirements and Use of the Framework

### 10.1. Define the Claim Before Selecting the Tests

Investigators should specify whether the intended claim concerns product competence, survival, intrinsic function, coupling, physiological rescue or visual restoration. Table 2 gives minimum evidence for each domain; these are claim-dependent criteria, not a requirement that every study complete every domain. A delivery study can be informative at M1, and a safety trial need not demonstrate donor-mediated visual restoration. The interpretation should remain within the domains actually tested.

The minimum common reporting set comprises product identity and relevant potency, donor authentication, topology, recipient disease stage and residual host substrate, follow-up, experimental unit, controls and complete recipient denominators. Negative, unmeasured and inconclusive findings should be distinguishable. The proposed minimum does not prescribe a universal cell dose, sample size or follow-up duration: these must match the precision, durability and biological claim under study. Multiple cell lines and manufacturing batches become important when generalisation beyond a single preparation is intended.

### 10.2. Feasible Core Evidence and Stronger Causal Tests

For RPE, the core functional test is an identified donor epithelial process operating at an appropriately oriented host interface. A shared M2/M3 observation is acceptable when its provenance, process and interface are resolved. Molecular polarity or static inclusions alone remain supportive. Transport, processing or retinoid assays should be chosen to match the proposed function rather than combined into a compulsory exhaustive battery. Donor-selective functional perturbation is a stronger attribution test where feasible.

For photoreceptors, the core evidence links donor light detection to a response in identified host retinas and includes an appropriate transmission control. For sheets, the same principle applies at the declared cell or product resolution: internal graft activity must be distinguished from passage across the host boundary. Cell-class-selective reporters, component changes, origin-resolved anatomy and pathway pharmacology offer complementary approaches. A viable donor product with selectively impaired phototransduction or release, or reversible donor silencing, offers stronger discrimination if survival and architecture remain comparable.

For RGCs, an afferent claim requires authenticated donor physiology dependent on host retinal input. An efferent claim adds continuous donor-pathway evidence, appropriate target innervation and functional transmission to central neurons. Donor-selective activation and recording at the target are more informative than eye illumination alone; reversible output suppression strengthens necessity attribution. Complete central reconstruction is currently a major technical barrier. That limitation justifies restricted afferent or anatomical claims, not relaxation of the requirements for a claim of restored donor output to the brain.

Some physiological evidence is obtained from donor-resolved explanted preparations. Such assays can test the function acquired after transplantation, but the preparation and terminal nature of the measurement must be stated; they do not establish continuous in vivo performance. Longitudinal optical or electrophysiological measurements can complement terminal mechanistic assays. Technical access and the proportion of recordable cells or eyes should be reported so that selected-cell proof of principle is not mistaken for overall treatment efficacy.

### 10.3. Choose Controls Proportionate to the Unresolved Mechanism

Controls should address the most plausible alternative for the product and assay. Active carriers may require carrier-only and cells-only comparisons; disruptive surgery requires procedure controls; residual host function requires host-specific measurements; and trophic alternatives may be informed by functionally impaired but viable donors. A complete factorial design is not feasible or necessary for every experiment. The report should state which alternative each control addresses and which remain unresolved.

Dead-cell and conditioned-medium comparators have limitations because inflammation and sustained secretion differ from living grafts. Genetic or electrical perturbations are also imperfect if they alter cell burden, health, tissue architecture or host preservation. Strong attribution requires convergent evidence rather than a prescribed number of controls. Reversibility is particularly useful when technically achievable, but destructive donor-ablation experiments should not be treated as a minimum requirement in humans.

### 10.4. Report a Profile Rather than a Highest Level

Each central claim should be accompanied by its mechanistic domains, separate outcomes, causal confidence and contextual limitations. The four illustrative applications in Table 4 show why an anatomical success, local physiological coupling, a component-specific causal result and incomplete RGC integration require different descriptions. The complete landmark-study matrix is retained in Supplementary Table S2, together with the original study denominators and methodological qualifications.

This reporting approach makes missing evidence visible without equating it with biological failure. It also separates a proposed experimental target from a demonstrated result. Independent testing of the framework’s clarity and reproducibility is needed before it could support formal consensus or standard-setting. Its immediate value is narrower: helping investigators and readers state what a study establishes, what remains uncertain and which feasible experiment would most directly resolve that uncertainty.

## 11. Conclusions

Retinal transplantation supports selected examples of donor survival and local functional coupling, but these achievements should be distinguished from donor-mediated visual restoration. RPE studies demonstrate feasibility and, in selected tissue, secure long-term donor persistence and supportive epithelial-interface activity. The strongest photoreceptor studies show that donor grafts can recruit host retinal pathways in severely degenerated experimental retina. Composite sheets provide evidence of reconstructed local circuits while making attribution to individual donor populations more demanding. RGC studies demonstrate afferent input to a small responsive subset, without establishing the complete functional central projection.

The proposed framework separates mechanism, outcome and causal confidence. Its domains are interpreted according to the cell product and claim, with overlapping RPE evidence and composite graft functions described explicitly. Unmeasured or inconclusive domains identify uncertainty; they do not demonstrate that replacement failed. Strong negative evidence requires a test capable of discriminating the proposed mechanism under the stated conditions.

Used as an interpretative and reporting aid, the framework can guide proportionate experiments and precise terminology without becoming an unvalidated regulatory threshold. Clinical benefit remains important when mechanism is unresolved, while claims of replacement require credible evidence that living donor cells perform the intended role through the appropriate host interface.

## Figures and Tables

**Table 1:** Operational terminology for retinal cell transplantation.

Term	Operational Meaning	Limit of the Claim
Donor survival	Viable donor-origin cells persist at a stated time.	Requires secure provenance; does not establish function.
Engraftment	Report survival, topology and host interaction separately.	An unqualified composite term is ambiguous.
Appropriate localisation	Position and orientation suit the intended epithelial or neural role.	Anatomical evidence alone.
Anatomical integration	Structural incorporation, neurites or candidate synaptic contacts.	Does not demonstrate physiological transmission.
Intrinsic functional maturation	Donor-resolved physiological performance after transplantation.	Markers and pre-transplant potency do not establish this.
Functional coupling	Physiological interaction across the required donor–host interface.	Epithelial for RPE; synaptic for neuronal products.
Functional rescue	Improvement or preservation after treatment.	May arise through replacement or other mechanisms.
Functional replacement	Donor cells perform the intended role through the appropriate host interface.	Requires at least moderate causal confidence under this proposed framework.
Physiological restoration	Recovery of retinal or pathway physiology attributable to the graft mechanism.	An objective response alone does not establish its source.
Visual restoration	Durable, meaningful visual recovery attributable to the graft.	An uncontrolled visual change is insufficient.

**Table 2:** Proposed minimum evidence by domain and practical limitations. These are claim-dependent evidential criteria, not validated regulatory or clinical-progression requirements. Stronger necessity tests are discussed in Section 10.

Domain	Minimum Evidence for the Stated Domain	Feasibility and Interpretation
M0 Product competence	Identity, purity, viability, maturation and relevant potency of the administered product; report safety-relevant composition.	Pre-transplant testing is feasible; sampled tissue may not represent every part of a sheet. It does not establish post-transplant function.
M1A Provenance and survival	Donor-specific nuclear, genomic or equivalent lineage evidence linked to intact surviving cells at a stated interval.	Often accessible in preclinical tissue; human imaging alone usually cannot authenticate living donor cells.
M1B Topology	Quantify position, orientation and coverage appropriate to the intended function, including misplaced tissue.	Imaging and histology are complementary. Correct graft-associated structure can be reported while provenance remains unresolved.
M2 Intrinsic function	A donor-resolved physiological assay of the intended intrinsic task after transplantation, with identity and stimulus controls.	Report in vivo versus explanted measurement. RPE processing/transport, photoreceptor light detection and RGC excitability require different assays.
M3-RPE Epithelial interface	Secure donor identity and polarity plus evidence that a specified epithelial process operates across the host interface.	A donor-resolved functional processing or transport assay may also inform M2; do not double-count it. Static inclusions are supportive.
M3-PR Photoreceptor transmission	Link donor light detection to an identified host retinal response, with spatial/temporal concordance and a discriminating transmission control.	Pharmacology helps but is not donor-specific. Residual-host tests and viable functionally impaired donors strengthen attribution.
M3-composite Sheet or organoid	Define the active unit and demonstrate physiological passage from authenticated donor tissue to host circuitry.	Cell-class attribution requires further dissection. Internal donor–donor activity or an isolated downstream response is insufficient.
M3-RGC-A Retinal input	Authenticated donor RGC with appropriate access to input layers and a physiological response dependent on host synaptic transmission.	Direct recordings or validated optical responses are feasible in selected cells; report survival and recording denominators.
M3-RGC-B Central output	Continuous donor-pathway evidence, appropriate target innervation and functional transmission to central neurons.	Long-distance reconstruction is a major barrier. Donor-selective stimulation strengthens source attribution; test retinotopy for organised-output claims.
O4A/O4B Retinal/pathway physiology	Objective response at the stated retinal or downstream site, with baseline or control comparison and defined spatial and temporal sampling.	Record separately from mechanism. A restoration claim additionally requires credible donor attribution; a null global test may miss local function.
O5A Animal behaviour	Prespecified, reproducible visually guided performance, masked assessment, appropriate controls and recipient-level analysis.	Report all attempted recipients and responder selection. Behaviour does not identify the cellular mechanism.
O5B Human visual benefit	Prespecified, clinically meaningful and durable visual-function change with stable baseline assessment and a credible counterfactual.	Direct donor perturbation is generally unsuitable. Clinical benefit and donor-mediated visual restoration require distinct interpretations.

**Table 3:** Assay groups and their principal inferential limits. Detailed safeguards are provided in Supplementary Table S1.

Assay Group	What It Can Support	Principal Inferential Limit
Lineage histology and imaging	Donor identity when specific; structure, location and persistence.	Reflectance, pigment or transferable labels do not authenticate donor physiology.
Molecular and ultrastructural assays	Cell phenotype, maturation and candidate interfaces.	Physiological performance and transmission remain unmeasured.
Donor-resolved physiology	Intrinsic function or host input/output, according to the design.	Selected cells do not establish treatment-wide success.
Retinal electrophysiology	Retinal activity; focal assays add spatial concordance.	Residual or preserved host retina can generate the signal.
Downstream recordings	Activity beyond the retina.	Does not identify donor versus host pathway.
Behaviour and clinical tests	Organism-level performance or patient benefit.	Does not independently establish donor replacement.
Donor-selective perturbation	Participation, necessity or sufficiency for a specified response.	Interpretation fails if graft health or host exposure also changes.

**Table 4:** Illustrative applications of the framework. Full study-level evidence and causal interpretations are retained in Supplementary Table S2.

Example	Supported Conclusion	Remaining Limit
RPE: Kashani et al. [15]	Donor-specific HLA evidence established two-year survival and polarity; inclusions supported interface activity.	No direct transport or retinoid-cycle measurement; acuity remained count-fingers.
Cones: Ribeiro et al. [17]	Lineage, local physiology, pathway blockade and a *CNGB3* donor comparator converged on donor-mediated coupling.	A restricted mouse-graft claim; no direct donor silencing or demonstrated human efficacy.
Organoid: Watanabe et al. [67]	Component and host-cell manipulations resolved contributions within a composite microcircuit.	Strong evidence for a restricted contribution does not authenticate every step of overall replacement.
RGC: Aguzzi et al. [20]	Synaptically dependent responses in an authenticated donor subset supported afferent integration.	Central output reconstruction and donor-mediated visual benefit were not demonstrated.

## Data Availability

No new data were created or analysed in this study. Data sharing is not applicable to this article.

## Supplementary Materials

The following supporting information can be downloaded at: https://www.mdpi.com/article/10.3390/cells15201840/s1, Section S1: Search strategy and evidence extraction; Section S2: Detailed assay interpretation; Section S3: Landmark studies and claim-specific interpretation; Section S4: Additional primary-study details; Table S1: Detailed assay capabilities, limitations and safeguards; Table S2: Complete landmark and mechanistic-study matrix.

## Abbreviations

AMD	Age-related macular degeneration
ERG	Electroretinogram
ESC	Embryonic stem cell
GMP	Good manufacturing practice
hESC	Human embryonic stem cell
hiPSC	Human induced pluripotent stem cell
HLA	Human leucocyte antigen
ILM	Internal limiting membrane
IPL	Inner plexiform layer
iPSC	Induced pluripotent stem cell
JAK	Janus kinase
L-AP4	L-2-amino-4-phosphonobutyric acid
LCA	Leber congenital amaurosis
MHC	Major histocompatibility complex
NAD(P)H	Reduced nicotinamide adenine dinucleotide (phosphate)
NMDA	N-methyl-D-aspartate
OCT	Optical coherence tomography
PSC	Pluripotent stem cell
RCS	Royal College of Surgeons
RGC	Retinal ganglion cell
RPE	Retinal pigment epithelium
RPESC-RPE	Retinal pigment epithelial stem cell-derived retinal pigment epithelium
VEGF	Vascular endothelial growth factor
M0	Product identity and competence
M1A	Donor provenance and survival
M1B	Appropriate graft topology
M2	Intrinsic donor function after transplantation
M3	Host–graft functional coupling
M3-RPE	RPE epithelial interface
M3-PR	Photoreceptor transmission to host retina
M3-composite	Composite graft–host coupling
M3-RGC-A	RGC retinal afferent input
M3-RGC-B	RGC central efferent output
O4A	Retinal physiology
O4B	Downstream visual-pathway physiology
O5A	Animal visual behaviour
O5B	Human visual benefit
