# Supplementary material for: From Engraftment to Vision: Defining Evidence of Functional Success in Retinal Cell Replacement Therapy
Source: Cells. 2026 Oct 10;15(20):1840. doi: 10.3390/cells15201840 (PMC13702508; doi:10.3390/cells15201840)
Supplement: Supplementary file 1 [file cells-15-01840-s001.zip › cells-4596439-supplementary.pdf]

Supplementary Material

From Engraftment to Vision: Defining Evidence of Functional Success in Retinal Cell Replacement Therapy

Reference numbers correspond to the unchanged reference list in the main manuscript.

S1 Search strategy and evidence extraction

The searches below were executed on 31 August 2026 without a language restriction at retrieval. Date limits were applied to each contemporary block. Targeted searches without a lower date limit were used only for seminal and mechanistic-sentinel studies.

RPE: (("retinal pigment epithelium"[Title/Abstract] OR "retinal pigment epithelial"[Title/Abstract] OR RPE[Title/Abstract]) AND (transplant*[Title/Abstract] OR graft*[Title/Abstract] OR implant*[Title/Abstract] OR replac*[Title/Abstract]) AND ("stem cell"[Title/Abstract] OR "stem cells"[Title/Abstract] OR pluripotent[Title/Abstract] OR "embryonic stem cell"[Title/Abstract] OR "embryonic stem cells"[Title/Abstract] OR "induced pluripotent stem cell"[Title/Abstract] OR "induced pluripotent stem cells"[Title/Abstract] OR iPSC[Title/Abstract] OR hESC[Title/Abstract])) AND ("2020/01/01"[Date - Publication] : "2026/08/31"[Date - Publication])

Photoreceptor: ((photoreceptor*[Title/Abstract] OR "rod precursor"[Title/Abstract] OR "rod precursors"[Title/Abstract] OR "cone precursor"[Title/Abstract] OR "cone precursors"[Title/Abstract]) AND (transplant*[Title/Abstract] OR graft*[Title/Abstract] OR replac*[Title/Abstract]) AND (retina*[Title/Abstract] OR subretinal[Title/Abstract])) AND ("2020/01/01"[Date - Publication] : "2026/08/31"[Date - Publication])

Retinal sheet/organoid: (("retinal organoid"[Title/Abstract] OR "retinal organoids"[Title/Abstract] OR "retina organoid"[Title/Abstract] OR "retina organoids"[Title/Abstract] OR "retinal sheet"[Title/Abstract] OR "retinal sheets"[Title/Abstract] OR "neural retina sheet"[Title/Abstract] OR "neural retina sheets"[Title/Abstract]) AND (transplant*[Title/Abstract] OR graft*[Title/Abstract] OR implant*[Title/Abstract])) AND ("2020/01/01"[Date - Publication] : "2026/08/31"[Date - Publication])

RGC: (("retinal ganglion cell"[Title/Abstract] OR "retinal ganglion cells"[Title/Abstract] OR RGC[Title/Abstract] OR RGCs[Title/Abstract]) AND (transplant*[Title/Abstract] OR repopulat*[Title/Abstract] OR replac*[Title/Abstract] OR graft*[Title/Abstract])) AND ("2020/01/01"[Date - Publication] : "2026/08/31"[Date - Publication])

S1.1 Retrieval and extraction details

PubMed was used as the sole structured bibliographic database. Searches were last run on 31 August 2026. Four independent searches covered RPE replacement, photoreceptor transplantation, retinal sheets or organoids, and RGC transplantation. The contemporary interval was 1 January 2020 to 31 August 2026, with no language restriction at retrieval. The searches returned 306 RPE, 512 photoreceptor, 130 retinal sheets or organoids, and 254 RGC records. Because records could enter more than one block, these counts are not additive. Deduplication by PMID yielded 914 unique records, comprising 254 review records and 660 non-review records. The exact search strings are reported above.

Seminal pre-2020 studies and mechanistic sentinels were added through targeted PubMed title, author and PMID searches seeded from high-overlap reviews and the reference lists of included primary reports. Each citation was verified against PubMed, the DOI record, the primary publisher or supplied full text. A verified PMID or DOI established bibliographic identity but was not treated as methods-level access. When complete primary text could not be obtained, extraction was restricted to facts reported in an authoritative abstract and the source limitation was retained.

Eligible evidence comprised primary human or in vivo animal studies of stem- or progenitor-derived products intended to replace RPE, photoreceptors, neuroretinal tissue or RGCs; mechanistic studies directly testing donor–host coupling, donor identity, material transfer, graft necessity or synaptic transmission; early clinical studies reporting graft persistence, objective retinal function or visual outcomes; and recent reviews used for overlap mapping and citation discovery. RPE suspensions, strips, monolayers, sheets and scaffold-supported constructs were included. Photoreceptor precursors, enriched rods or cones, dissociated organoid-derived cells,

retinal sheets and organoid grafts were included. RGC transplantation was retained as the paradigm with the most demanding reconstruction requirement.

Mesenchymal stem cell (MSC) and stromal-cell treatments intended principally for paracrine or neuroprotective action, extracellular vesicles, endogenous Müller-cell reprogramming, gene therapy, optogenetics and retinal prostheses were excluded as primary subjects. They were considered only when necessary to distinguish replacement from alternative routes to rescue. Autologous retinal free flaps, amniotic membrane grafts, mitochondria transplantation, whole-eye grafts and non-retinal tissues were also excluded. In vitro differentiation studies were retained only when they directly informed product competence or a transplantation claim.

For each analytical study, we extracted citation and year, cell product, recipient disease or model, species or population, delivery method, follow-up, donor survival and localisation, maturation, anatomical integration, functional or synaptic coupling, objective physiology, behavioural or clinical outcome, evidence supporting donor-cell causality, assessment of material transfer or paracrine effects, residual host function, proposed evidence coordinates and major methodological limitations. Experimental unit, masking, randomisation, sham or carrier controls, responder selection, attrition and the distinction between peak and terminal outcomes were recorded when reported.

The structured map contained 37 overlapping reviews, 73 contemporary priority primary or mechanistically important records, and 14 verified pre-2020 landmarks. A 79-study analytical corpus underwent structured evidence adjudication: 71 studies were assessed at full-text or methods level and eight remained partial-primary or abstract-level after final source reconciliation. The latter were not used to support unreported denominators, controls or method-specific inferences. The evidence architecture was calibrated iteratively against representative RPE, photoreceptor, retinal-sheet and RGC studies rather than imposed unchanged on the literature.

S2 Detailed assay interpretation

Table S1. Detailed assay capabilities, limitations and safeguards. Safeguards are selected according to the proposed claim and feasibility; they are not a compulsory checklist for every study.

Assay	What it can support	What it cannot establish alone	Safeguards and stronger tests where feasible
Donor-lineage histology	M1A donor provenance and survival; M1B when anatomy and orientation are also shown.	Longitudinal viability, mature physiology, host coupling or contribution to an outcome. Cytoplasmic and pigment labels may be transferred, ingested or host derived.	Combine a non-transferable nuclear or genomic lineage marker with morphology, compartment and a viability-compatible readout.
OCT, fundus autofluorescence and adaptive-optics imaging	Implant position, graft-associated structure, retinal thickness and longitudinal topological change.	Living donor-cell identity, donor physiology or the cellular source of reflectance, pigmentation and lamination.	Co-register imaging with donor-specific tissue evidence where available and report treated, adjacent and untreated retinal loci.
Marker expression, transcriptomics and ultrastructural maturation	Product characterisation (M0) and maturation phenotype; not donor-autonomous physiological function (M2).	Performance of the inferred function in vivo. A molecular resemblance to native cells is not a physiological measurement.	Pair with donor-resolved post-transplant function and quantify abnormal as well as native-like states.
RPE outer-segment inclusions or phagosome markers	Contact with host outer segments and supportive M3-RPE interface activity when donor provenance is secure.	Sustained ingestion, lysosomal processing, retinoid-cycle function, vectorial transport or clinically relevant photoreceptor support.	Use pulse-chase or processing assays, donor-specific localisation and longitudinal host-function measurements.
Synaptic-marker apposition or electron microscopy	Candidate anatomical contacts and increased plausibility of M3.	Signal direction, sign, strength, receptive-field organisation or contribution to a retinal response.	Link origin-resolved contacts to donor-recorded or host-recorded transmission with pathway-selective perturbation.

Assay	What it can support	What it cannot establish alone	Safeguards and stronger tests where feasible
Donor patch clamp, calcium or voltage imaging	M2 if an intrinsic donor response is isolated; M3 when a securely identified donor response depends on host synaptic input or drives host output.	Population-wide integration, downstream pathway reconstruction or visual benefit. Calcium signals do not necessarily establish spike output.	Record all attempted units and eyes, verify the recorded cell's lineage, map the stimulus and use appropriate synaptic or donor-specific perturbation.
Full-field, focal or micro-ERG	O4A retinal physiology; focal methods improve spatial concordance with a graft.	The cellular source of recovery. Full-field signals can arise from residual host retina; even focal signals may reflect protected host cells.	Compare graft and matched non-graft loci, document residual host cells and test whether donor function is necessary for the response.
Superior-colliculus, visual-evoked or cortical responses	O4B downstream pathway activity.	Whether donor photoreceptors or RGC axons carried the signal when any host visual pathway remains.	Use donor-selective activation and reversible silencing, latency and retinotopic mapping, plus continuous donor-pathway tracing.
Optomotor and other visually guided behaviour	O5A organism-level visual rescue when analysis is masked and animal-based.	Cell replacement, donor necessity or the exact retinal pathway used. Learning, non-visual cues and residual islands can contribute.	Pre-specify success criteria, include every treated animal and repeat sessions with sham controls and donor-off testing where feasible.
Visual acuity, microperimetry, fields and patient-reported function	O5B clinical benefit; microperimetry can add treated-field localisation.	Living donor survival or donor-mediated mechanism. Surgery, learning, fixation, natural history and co-interventions may change performance.	Use a credible concurrent counterfactual, stable baselines, masked assessment, retinal-locus tracking and a prespecified clinically meaningful threshold.
Donor-specific activation, silencing or ablation	Causal sufficiency or necessity for a defined physiological or visual claim.	A clean mechanism if the perturbation also changes graft survival, trophic output, inflammation or tissue architecture.	Demonstrate reversibility where possible and show that donor amount, localisation, viability and host reserve remain otherwise comparable.

S3 Landmark studies and claim-specific interpretation

Table S2. Complete landmark and mechanistic-study matrix. "Not demonstrated" identifies an evidential limit unless an adequately measured negative finding is explicitly reported. Causal confidence applies to the named claim, not to the study as a whole.

Study and context	Strongest justified mechanistic evidence	Outcome evidence	Claim-specific causal interpretation
RPE - Liu et al., 2021 [13]	Human RPE stem-cell-derived monolayer on PET in a surgically prepared non-human-primate submacular bed; three months. M1A/M1B were demonstrated; polarity and outer-segment uptake supported M3-RPE but did not directly establish donor transport or retinoid-cycle function.	Full-field ERG was unchanged; O4 restoration was not shown and no O5 endpoint was assessed.	Grade B for the restricted interface claim. Scaffold-only and sham controls were valuable, but small groups and a healthy surgically created recipient bed limit inference about efficacy in chronic degeneration.
RPE - Parikh et al., 2023 [14]	Stem-cell-derived RPE recovered from rabbit subretinal grafts at one month. Secure donor identity and topology supported M1A/M1B; single-cell transcriptomic movement towards adult RPE supported molecular maturation; donor-autonomous physiology (M2) was not assessed.	No O4 or O5 restoration endpoint was assessed.	Grade B for maturation biology. A short healthy-recipient experiment and cell-level trajectory analysis cannot establish physiological epithelial replacement.

Study and context	Strongest justified mechanistic evidence	Outcome evidence	Claim-specific causal interpretation
RPE - Kashani et al., 2022 [15]	Two-year postmortem analysis of one bioengineered hESC-RPE implant in advanced geographic atrophy. Donor–recipient HLA mismatch securely established M1A/M1B; polarity and rhodopsin-positive inclusions supported, but did not directly prove, M2/M3-RPE.	No O4 restoration; acuity remained count-fingers without O5B benefit.	Grade A for donor survival and grade B for marker-based epithelial activity. The single end-stage case lacked donor-resolved physiology and viable overlying photoreceptors.
RPE - Humayun et al., 2024 [40]	Long-term follow-up of 15 implanted participants with advanced geographic atrophy. Imaging established durable implant localisation (M1B), but not living donor-cell provenance, M2 or M3-RPE.	Inter-eye BCVA trajectories provided an exploratory O5B association only.	Grade C for donor-mediated efficacy. The safety-designed single-arm study was vulnerable to baseline inter-eye asymmetry, residual host retina, surgery, learning and natural variability.
RPE - Kesavamoorthy et al., 2026 (online 2025) [16]	Nine-year postmortem analysis after subretinal hESC-RPE suspension in one person with advanced Stargardt disease. Graft-site pigmented RPE-like tissue supported M1B, but donor lineage was not secured; M2, M3-RPE and O4 were not demonstrated.	No visual improvement; ex vivo FLIM was neither donor-cell resolved nor an in vivo O4 endpoint.	Grade B for long-term graft-site anatomy and grade C for donor survival or replacement. Host-RPE hyperplasia, a single biological unit and region-level pseudoreplication prevent stronger attribution.
Photoreceptor - Pearson et al., 2012 [47]	Post-mitotic rod precursors in murine degeneration with residual host photoreceptors. Reporter-positive host-outer-nuclear-layer cells were originally interpreted as integration; current provenance standards leave M1A insecure.	Retinal, downstream and behavioural rescue were reported (O4/O5A).	Retrospective grade C for donor replacement. Later material-transfer evidence means the outcomes cannot be assigned securely to newly integrated donor photoreceptors.
Photoreceptor mechanism - Pearson and Santos-Ferreira et al., 2016 [10, 11]	Independent dual-lineage studies showed that host photoreceptors can acquire donor reporters, proteins and cytoplasm while retaining host nuclei. These are mechanism sentinels, not attained replacement coordinates.	No O4 or O5 efficacy claim was required.	Grade A for donor–host material transfer. The studies invalidate transferable reporters as sole provenance evidence but do not establish that transfer dominates every product or disease context.
Photoreceptor - Ribeiro et al., 2021 [17]	Purified human PSC-derived cones in advanced rd1 degeneration. Secure human nuclear identity, local physiology, a non-functional *CNGB3* donor control and reversible glutamatergic blockade supported M3-PR.	Local retinal physiology and visually guided behaviour supported O4A/O5A.	Grade A for the replacement claim tested. It remains a mouse xenograft without direct donor silencing, but the convergent specificity controls make generic trophic rescue insufficient.
Photoreceptor - Procyk et al., 2025 [18]	Human cones in end-stage AIPL1-deficient mice at ten weeks. Graft-centred micro-ERG supported M2; host-RGC responses and reversible pathway blockade supported M3-PR.	O4A was demonstrated locally; full-field ERG was negative and optomotor thresholds were measurable in 8/15 treated eyes with incomplete repeatability.	Grade A for restricted M3-PR/O4A and grade B for O5A. Retina-level samples were small and donor necessity was not tested.
Photoreceptor - Procyk et al., 2026 [54]	Human cones transplanted into 12–15-month-old rd1 mice after prolonged photoreceptor loss. Graft-localised responses and host inner-retinal processing supported M3-PR despite advanced remodelling.	All four technically successful MEA preparations showed O4A; eye-specific behaviour was measurable in 5/9 treated eyes.	Grade B. The stringent model strengthens relevance, but conditioning on technically successful retinas, unmasked MEA analysis and absence of donor-off testing constrain generalisability.
Retinal sheet - Seiler et al., 2010 [68]	Embryonic rat retinal progenitor sheets in S334ter degeneration. Donor processes, candidate ultrastructural synapses and graft-field concordance supported M3 for the composite graft.	Superior-colliculus responses mapped to graft-related retinal locations (O4B).	Grade B. Selected animals with clear media, incomplete same-animal physiology-histology mapping, composite cell content and possible trophic effects preclude cell-specific replacement attribution.

Study and context	Strongest justified mechanistic evidence	Outcome evidence	Claim-specific causal interpretation
Retinal sheet - Mandai et al., 2017 [63]	Mouse iPSC-derived retinal sheets in end-stage rd1 mice. Genetic synapse labelling and host-RGC light responses supported M3-PR/composite coupling.	Focal retinal responses and visually guided behaviour supported O4A/O5A.	Grade B. OCT-based selection of good grafts, behavioural training and retinoid exposure, composite graft content and no donor-off experiment limit population-level causality.
Retinal sheet - Akiba et al., 2024 [65]	Reporter-equipped organoid sheets in rd1 mice. Array tomography linked candidate host–graft microstructures spatially with host-RGC light responsiveness, supporting M3-PR.	Graft-centred MEA responses supported O4A; no O5 endpoint.	Grade B. Synapse-response correlation is informative but not donor necessity; manual response classification, exclusions and electrode-level nesting remain concerns.
Retinal organoid - Watanabe et al., 2025 [67]	*Isl1*-null organoid sheets and host horizontal-cell depletion dissected a composite circuit. Serial ultrastructure and host-RGC responses supported M3-PR while identifying a graft-horizontal-cell contribution.	Graft-centred host-RGC responses supported O4A; no O5 endpoint.	Grade A for the restricted horizontal-cell contribution and grade B for overall replacement. Hundreds of cells were nested within 16 retinas, and donor necessity remained untested.
Retinal organoid - Hirami et al., 2023 [76]	Allogeneic iPSC-derived retinal sheets in two people with advanced retinitis pigmentosa. Two-year imaging supported M1B persistence; living donor provenance, M2 and M3 were not demonstrated.	A slower visual decline than in fellow eyes was exploratory; O5B was not established.	Grade C for functional replacement. Two participants, inter-eye asymmetry, residual host function and absence of donor-specific physiology confine the result to safety and graft-associated anatomy.
Retinal organoid - Ozaki et al., 2026 [75]	*ISL1*-null human organoids in an acute laser-induced macaque macular lesion, followed for up to two years. Host-bipolar contacts and focal physiology supported M3-PR.	Focal b-wave improvement supported O4A in 2/4 engineered-graft eyes; no meaningful visual-behaviour endpoint.	Grade B. Primate scale and durability are strengths, but the acute lesion, tiny cohort, graft loss/endophthalmitis, protocol changes and no donor-off test limit clinical extrapolation.
RGC - Venugopalan et al., 2016 and Vrathasha et al., 2022 [92, 93]	Primary/developing or hiPSC-derived RGCs in healthy adult rodent retina. Direct donor-cell light responses supported M3-RGC-A in 5 cells from 3 retinas and 4/6 recorded cells from 4 retinas, respectively; M3-RGC-B was not demonstrated.	No donor-attributed downstream physiology or visual behaviour (O4B/O5).	Grade B for restricted afferent input. Very small selected functional samples, low survival, healthy host circuitry and absent central reconnection prevent a replacement claim.
RGC - Zhang et al., 2021 [84]	Human stem-cell-derived RGCs on organotypic mouse retina. ILM proteolysis increased parenchymal entry and neurite growth, establishing a controlled M1B barrier mechanism.	No physiological M3, O4 or O5 endpoint.	Grade A for the ILM-barrier claim, not for replacement. A short ex vivo structural experiment cannot address in vivo survival, afferent input or retinofugal reconstruction.
RGC mechanism - Zhang et al., 2023 [91]	Human RGC xenografts after ILM disruption transferred donor-associated cytoplasmic, nuclear-associated and mitochondrial labels to host Müller glia. Species-specific nuclear organisation distinguished donor neurons from labelled host cells.	No M3, O4 or O5 efficacy endpoint.	Grade A for the provenance-confound claim. Events were rare and xenogeneic, but they make an isolated transferable marker insufficient for authenticating the small responsive subsets on which RGC claims may depend.
RGC - Aguzzi et al., 2026 [20]	Human PSC-derived RGCs across rodent, macaque and human-explant models. After ILM disruption, 73/2,553 donor cells in 28 retinas were light responsive versus 1/247 in 10 intact-ILM retinas; cobalt chloride blocked the responses. This supports M3-RGC-A only.	No M3-RGC-B, donor-attributed O4B or O5 endpoint.	Grade A for the restricted afferent-coupling claim. Only about 3% of donor cells responded, signals were weak or slow, observations were nested, and no functional central pathway was reconstructed.

S4 Additional primary-study details

The following details supplement the condensed cell-specific discussions. They retain design features, denominators and limitations needed to interpret the primary observations; repeated general framework recommendations are omitted.

S4.1 RPE products and studies

Recent constructs illustrate why carrier controls are essential. Microgels, synthetic membranes and degradable fibrin can alter cell survival, inflammatory access, fluid movement and the wound environment independently of living donor function. In minipig experiments, an acellular scaffold itself caused marked local degeneration, so relative preservation by a cell-containing patch supported a product effect but did not isolate epithelial replacement. In a porcine debridement model, a fibrin-supported monolayer was associated with photoreceptor and choriocapillaris preservation, but only four animals were used, follow-up was one to two months, inflammatory changes were common and there was no concurrent acellular-fibrin arm. Microgel-supported RPE in RCS rats likewise improved some structural and electroretinographic measures, but transferable DiI labelling, residual host photoreceptors and trophic activity of both cells and carrier left donor-RPE causality unresolved [26–28]. A defensible design requires, where feasible, living functional cells, non-functional or dead cells, carrier alone, surgery alone and untreated disease controls.

RPE grafts are unusually susceptible to false attribution because several host processes generate graft-like signals. Host RPE can migrate or become hyperplastic; macrophages can accumulate melanin; cytoplasmic dyes can fade or transfer; and OCT hyperreflectivity can persist after loss of the intended monolayer. Clinical fundus pigmentation and implant thickness are consequently evidence of graft-associated anatomy, not secure M1A donor survival. Human-specific nuclear or genomic markers in xenografts are more persuasive, while donor–recipient HLA discordance can provide particularly strong human postmortem provenance. In one participant examined two years after implantation, reciprocal HLA staining, RPE65/BEST1 expression and apical Na^+/K^+-ATPase established surviving donor RPE on the parylene membrane much more securely than imaging alone [15].

Long follow-up cannot repair weak lineage evidence. Five-year imaging after a human embryonic stem cell (hESC)-RPE patch showed persistent pigmentation and implant-associated thickness, but living donor identity was not available and pigmented macrophages or host RPE could not be excluded [29]. The nine-year postmortem Stargardt report is even more instructive. Pigmented RPE-like tissue occupied the graft-associated superior macula, with both monolayer and multilayer regions, but no donor-specific nuclear, genomic or sex-chromosome assay was obtained. The untreated eye contained host RPE hyperplasia, demonstrating the biological plausibility of the principal alternative. The correct conclusion is long-term persistence of graft-site pigmented RPE-like tissue, not proven nine-year survival of donor hESC-RPE [16]. This distinction is consequential because all subsequent claims of donor maturation or metabolism depend on M1A.

Molecular phenotype and morphology are necessary but insufficient. Single-cell transcriptomics has shown that transplanted stem-cell-derived RPE can move towards an adult native expression state, but trajectory towards maturity is not measurement of epithelial performance [14]. Correct apical-basal marker distribution, tight-junction proteins, microvilli, basal infoldings and collagen-IV deposition similarly support competence and topology. Rhodopsin-positive inclusions within donor-proven RPE provide stronger interface evidence because they are consistent with uptake of host outer segments. Such observations have been reported in mouse and non-human primate grafts and in the HLA-mismatched human implant [13, 15, 24, 30]. Nevertheless, a static inclusion does not by itself establish the rate, completeness or durability of outer-segment processing, nor does it test transport, barrier or visual-cycle function.

Large-animal studies answer a different question. Macular implantation in non-human primates has demonstrated that preformed human RPE monolayers can be placed in the intended compartment, retain polarity and phagocytose host outer-segment material, although favourable structural outcomes occurred in only a subset and follow-up was short [13, 24]. Human iPSC-RPE strips survived and expanded for up to six months at laser-injured and intact primate sites, but did not significantly preserve outer-nuclear-layer thickness relative to laser-only regions, and no physiological endpoint was tested [25]. Immune-evasion studies have extended anatomical persistence in healthy primate eyes, and ex vivo JAK inhibition has been reported to reduce immune attack on RPE grafts; these studies identify mechanisms of M1 survival, not M2/M3-RPE function [33, 34]. Acute

debridement, healthy Bruch's membrane and surgically prepared recipient beds are appropriate for delivery and safety questions but cannot reproduce chronic geographic atrophy, aged matrix, choriocapillaris loss or advanced retinal remodelling.

The earliest hESC-RPE suspension studies established that a pluripotent-cell-derived product could be placed subretinally without early evidence of uncontrolled proliferation, and later follow-up described persistent pigmentation and visual-acuity changes in some eyes [35, 36]. These were important translational achievements, but the studies were open-label, small and designed for safety. Pigmentation was not donor-specific physiology, treated eyes retained variable host retina, and letter gains were vulnerable to learning, regression to the mean and inter-eye asymmetry. The first autologous iPSC-RPE sheet recipient had graft persistence and stable acuity after removal of a neovascular complex, but one treated patient and extensive surgery cannot establish efficacy [37]. Similarly, the two-patient hESC-RPE patch study reported improved acuity, reading speed and fixation after surgery for severe haemorrhagic neovascular AMD; haemorrhage removal, vitrectomy, anti-VEGF treatment and residual host tissue remained inseparable from any donor-cell effect [38].

Later small clinical series do not overcome this limitation. Five-year follow-up of two patch recipients showed divergent visual trajectories: one retained a ten-letter gain, whereas the other fell below baseline, despite persistent graft-associated imaging [29]. Three recipients of iPSC-RPE strips had localised graft-associated anatomy but no consistent microperimetric or clinically meaningful visual improvement, and immune reaction, epiretinal membrane and oedema complicated interpretation [41]. In a six-participant low-dose RPESC-RPE suspension cohort, a large mean gain was reported in the three worse-seeing eyes at 12 months, whereas the three better-seeing eyes had shorter follow-up and a much smaller mean change; failed microperimetry, absence of an imaging marker for the non-pigmented cells and lack of a concurrent control make the subgroup contrast particularly susceptible to baseline asymmetry and regression to the mean [42]. These signals justify controlled testing, not an efficacy claim.

Rare-disease and safety series further illustrate the need for a stable counterfactual. In one *RPE65*-associated LCA recipient, most transplanted cells migrated into an epiretinal membrane, intraocular pressure reached 46 mmHg, vision transiently fell to light perception, and subsequent functional changes occurred in both treated and untreated eyes after several interventions [43]. In a phase I Stargardt study, acuity change was non-significant and some eyes underwent concurrent cataract surgery [44]. Three-year follow-up of three Asian Stargardt recipients described one improved and two stable eyes, but relied on fellow-eye and external natural-history comparison [45]. Such reports remain valuable for adverse-event detection, surgical learning and hypothesis generation. They cannot show that living donor RPE caused the visual course.

Postmortem analysis can bridge the otherwise inaccessible interval between clinical imaging and cell identity, but its inference depends on the markers available. The HLA-mismatched parylene case demonstrated donor origin, correct polarity and outer-segment uptake at two years, yet provided no direct transport or retinoid-cycle measurement and no visual benefit [15]. The nine-year Stargardt case provided exceptional temporal and spatial concordance, but donor lineage was not established. Its fluorescence-lifetime NAD(P)H measurements were obtained after fixation and deparaffinisation, were not cell resolved, and showed the same macula-versus-periphery direction in both eyes. Treating eight sampled regions per location as independent biological replicates also overstates the information supplied by one person and one eye [16]. Metabolic difference at a graft site is therefore neither M2 nor O4 unless it is donor-specific, functionally interpretable and analysed at the correct experimental level.

S4.2 Photoreceptor studies

For a dissociated product, M1B requires more than residence in the subretinal space. The apical pole must face the RPE and interphotoreceptor matrix, the basal pole must have access to host bipolar or horizontal neurites, and rosettes, reflux, epiretinal migration and isolated cell clumps must be quantified. Large-animal studies illustrate the gap between delivery and replacement. Human iPSC-derived photoreceptors could be followed for up to 16 weeks in the cone-dominant ground squirrel, with human nuclear antigen and lamin B2 providing secure xenograft provenance, but retention was variable and cells also occupied vitreous, epiretinal and intraretinal compartments [49]. In dogs, reporter-positive cells co-labelled with human Ku80 and survived for up to 22 weeks under systemic immunosuppression, yet synaptophysin-positive pedicle-like structures and bipolar-dendrite

apposition remained anatomical candidates rather than transmitting synapses [50]. Both studies materially advance M1A and delivery biology; neither demonstrates M3-PR.

Longitudinal fluorescence and OCT are valuable for locating graft-associated tissue, estimating coverage and detecting adverse anatomy. They cannot establish that every imaged cell is alive, donor-derived or physiologically competent. In a study designed specifically to develop imaging biomarkers, structural grades correlated with terminal histology in a subset of eyes, but no eye improved in grade and no donor or host physiological outcome was measured [51]. Imaging should therefore be reported as M1B evidence unless it is paired with a validated viability reporter and donor-resolved functional assay. The same constraint applies when a graft becomes smaller, brighter or more laminated: each change has several biological explanations.

Photoreceptor ribbons, bassoon, synaptophysin, pikachurin and apposed bipolar dendrites are appropriate anatomical observations, but colocalisation at optical resolution does not establish membrane specialisation, vesicle release, postsynaptic responsiveness, direction of signalling or necessity. Pikachurin-associated puncta after transplantation into late-stage rd1 retinas illustrate this boundary: the molecule was present at host–graft interfaces, but no ultrastructural or physiological transmission was tested [52]. Electron microscopy can strengthen the structural case by resolving pre- and postsynaptic specialisations, yet even a morphologically complete synapse may be silent or aberrant. Anatomy can identify where to test function; it cannot substitute for that test.

Several positive studies illustrate how an outcome can outrun its mechanism. In advanced degeneration, transplanted PSC-derived photoreceptor precursors were associated with light avoidance, optomotor responses and retinal electrophysiology, but 15 of 30 treated animals were first classified as behavioural responders and subsequent assays were concentrated in that selected group [55]. Donor identity was supportive and the responses were biologically interesting, yet conditioning on post-treatment response changes the denominator and weakens generalisability. Without a non-functional donor or donor-off contrast, the result remains compatible with a donor contribution but does not reach the causal standard of the strongest focal studies.

Pupillary constriction is still less specific. Dissociated cells from early chemically induced retinal organoids produced a pupil-response difference at some irradiances in rd10 mice, but the product was unsorted, donor provenance relied on a cytoplasmic reporter, host bipolar proximity was anatomical, and a stated full-field ERG comparison was not presented [56]. Residual host photoreceptors, intrinsically photosensitive ganglion cells, consensual pupil circuitry, trophic rescue and material transfer remain viable explanations. A pupil reflex can establish that afferent light signalling changed; it cannot establish image-forming vision or donor-photoreceptor necessity. The appropriate code is a transplantation-associated downstream response, not demonstrated M3-PR.

Composite products and early treatment create a different attribution problem. In sodium-iodate-injured mice, only co-transplantation of hESC-RPE and mouse photoreceptors in hydrogel met the prespecified ERG response criterion and improved scotopic behaviour; unusually strong component controls showed that the combination mattered [57]. Yet, donor rods were few and immature, physiology was not donor resolved, and RPE support, residual host photoreceptors and trophic cooperation remained plausible. In *Pde6b*-knockout rats treated before expected degeneration, a mixed hiPSC-derived RPE/photoreceptor-characteristic suspension preserved full-field ERG responses for up to nine months, but no component-specific function or donor-to-host transmission was shown [58]. Both studies support therapeutic rescue or preservation. Neither demonstrates reconstruction of a previously absent photoreceptor circuit.

Mechanistic studies of graft survival should be kept within their own claim boundary. Donor and host *Ripk3* perturbations, including bone marrow chimeras, showed that necrotic-cell-death and immune pathways can alter four-week precursor-graft survival [59]. The same work observed host photoreceptor protection after retinal detachment without transplantation, directly illustrating that neuroprotection can arise independently of donor replacement. These data are strong for a restricted survival mechanism and useful for improving M1. They do not establish M2, M3-PR or visual restoration.

S4.3 Retinal sheet and organoid studies

M0 is unusually consequential for self-organising tissue. In a translational manufacturing study, retinal differentiation also generated eye-related, cortex-like and spinal-cord-like tissues. A paired quality-control

strategy tested an outer ring from each aggregate by quantitative PCR while preserving the central cap for transplantation, and long-term tumourigenicity studies found no transplant-related lesion [60]. This is a thoughtful solution to destructive release testing, but it remains a surrogate: the assayed ring is not the implanted cap, and spatial heterogeneity can weaken the correlation between them. Release criteria should report the validation error of that inference, the detection limit for off-target lineages and the full denominator of manufactured sheets, not only those released.

Post-transplant composition can also diverge from pre-transplant appearance. Single-cell analysis of human organoid fragments placed into photoreceptor-deficient mice identified donor-derived astrocytes and brain/spinal-cord-like neural precursors that migrated through recipient retinal layers, whereas donor rods and cones largely remained subretinal and matured faster than time-matched cultured controls [61]. Human nuclear markers made the provenance claim persuasive, but the experiment did not determine whether the non-retinal populations were induced by the host or selected from rare pre-existing cells. Its importance is independent of efficacy: persistence of a tissue labelled as retinal can include biologically active non-retinal donor cells. M0 must therefore be linked to post-transplant M1A surveillance rather than treated as a one-time certificate.

Correlative array tomography has materially strengthened the anatomical case. In rd1 mice, reporter-resolved host–graft contacts reproduced ribbon-associated microstructures involving horizontal-cell processes followed by host rod-bipolar dendrites. A spatial analysis then related local candidate-synapse density to the number of light-responsive host ganglion cells recorded by multielectrode array [65]. This convergence is more persuasive than marker colocalisation alone and supports M3-PR with grade B confidence. It is not a necessity experiment: ultrastructure was examined in two animals, the physiology-imaging cohort comprised 12, response classification was operator-defined, and electrodes nested within a retina risk being treated as independent evidence. One wild-type graft sample also contained candidate synapses without responsive host ganglion cells, confirming that structure is not sufficient.

The quality of restored physiology is a separate outcome. Genome-edited organoid sheets in six rd1 retinas generated host ganglion-cell responses that showed light adaptation and some flicker following, rather than only a flash-evoked event [66]. Comparison with wild-type retina therefore asked whether the reconstructed circuit performed elementary visual computations. The answer was partial: transplanted responses remained slower or incomplete, few cells followed frequencies above 5 Hz and two grafted retinas had low responses attributed to technical problems. These data advance O4A by testing what the recovered signal can do. They do not directly measure donor-photoreceptor M2 or prove which donor–host edge generated it.

Older retinal-sheet work established the value of spatial concordance. Embryonic rat retinal progenitor sheets survived for months in S334ter rats, donor processes entered host retina, synapse-like profiles were seen by electron microscopy and superior-colliculus responses mapped to retinal locations corresponding to the graft [68]. The study included sheet variants and sham controls, but recordings were performed in 15 selected animals from a 21-rat cohort, clear ocular media were required, physiology and histology did not always come from the same animal, and neither masking nor randomisation was reported. It remains a landmark demonstration of graft-associated pathway rescue, not cell-specific proof for a contemporary pluripotent-stem-cell product.

Human ESC-derived organoid sheets subsequently survived for 54-300 days in immunodeficient retinal-degeneration rats and were associated with optokinetic and superior-colliculus responses [69]. Masked scoring and sham and age-matched controls strengthen the treatment association. Secure human markers and long follow-up strengthen survival. The mechanistic bridge remains incomplete: the graft contained multiple retinal cell types, candidate connectivity was mainly anatomical, residual host circuitry was not selectively silenced and trophic preservation could contribute. O4B and O5A can therefore be recorded without elevating the study to donor-photoreceptor-resolved M3.

Recipient pathology can make the alternative mechanism more plausible than the stated one. In RCS rats, whose primary defect is RPE phagocytosis rather than a photoreceptor-autonomous degeneration, hESC-derived organoid sheets were associated with an early full-field ERG advantage, optokinetic thresholds and superior-colliculus responses in seven of 14 tested grafted rats [70]. The ERG difference disappeared by four months, host cones were preserved near grafts and the tissue product could supply trophic support. Randomisation, masking and sham controls support transplant-associated rescue, but the model cannot distinguish replacement of lost photoreceptors from preservation of host cells exposed to an altered subretinal environment.

A shipping study usefully tested whether hESC organoid sheets retained biological activity after overnight transport [71]. Fifty-six rats underwent transplantation, whereas optokinetic analysis used a selected subset of 14 and superior-colliculus recording used five; four of those five showed responses, and one illustrative response map resembled the graft outline. This supports logistical feasibility and selected transplant-associated O4B/O5A. It does not establish point-to-point donor reconstruction across the cohort. Technical failures, misplaced or traumatic grafts and the reduction from the surgical denominator to the functional denominator must remain visible whenever efficacy is described.

Large eyes expose delivery, immune and topological problems that rodent studies cannot reproduce, but evidence must remain within the arm in which it was generated. MHC-homozygous monkey iPSC-derived retinal sheets survived for three to six months after matched or mismatched transplantation into laser-injured macaque retinas, with maturing rosettes and no overt clinical rejection; peripheral immune assays nevertheless suggested subclinical rejection in three of four mismatched animals [72]. Host–graft synapses were not clearly established and no restored physiology was shown in the monkeys. A separate mouse experiment detected host ganglion-cell responses, but those data cannot supply the missing M3 or O4 evidence in primates or isolate MHC effects across confounded strains and pathologies.

In Yucatan minipigs with focal cobalt-chloride outer-retinal injury, control and hypoimmunogenic iPSC-derived sheets formed an immature photoreceptor-like layer three to four nuclei thick [73]. Correctly oriented tissue in one eye coincided with local photopic multifocal-ERG improvement at days 45 and 60, whereas a folded marker-positive graft did not. The spatial and topological contrast is mechanistically valuable. It remains hypothesis-generating: only one eye supplied the positive physiology, outer segments were absent or immature, interface markers were anatomical, regional hexagons were nested within that eye and an acute chemical lesion differs markedly from chronic inherited degeneration.

A single-macaque macular-hole experiment provides an especially clear counterexample to equating improvement with neuronal replacement. A human PSC-derived sheet filled and closed the experimentally created defect and developed rod- and cone-marker-positive cells, while fixation and focal electrophysiology changed over approximately 200 days [74]. No host–graft synaptic connection was confirmed, the untreated fellow eye also improved as its lesion recovered, and the graft acted as a structural filler in an acute wound. The study supports surgical and anatomical feasibility. Any functional effect could arise from closure, tissue support, inflammation control, residual retina or spontaneous recovery; M3 was not demonstrated.

The first-in-human allogeneic iPSC-derived retinal-organoid sheet study was designed primarily for safety. Two people with advanced retinitis pigmentosa showed stable graft-associated structure and increased retinal thickness at the implantation site for two years without a serious graft-related adverse event [76]. Visual decline was described as less progressive than in untreated fellow eyes. With two non-randomised participants, biologically asymmetric eyes, substantial residual function and no donor-specific physiology, that trajectory cannot establish O5B. Imaging supports durable M1B; living donor provenance is inferred, and M2, M3 and O4 remain unproved.

Adaptive-optics optical coherence tomography (OCT) later resolved rosette-like structures and an outer-plexiform-layer-like reflective line in one of these patients at approximately two years and three months [77]. The images are useful for longitudinal surveillance and hypothesis generation, but reflectance cannot identify donor nuclei, demonstrate viable phototransduction or show signal transfer. Histological resemblance to a graft in a different cynomolgus monkey, produced in a different injury and transplantation context, cannot validate the cellular identity or function of the human feature. This is high-resolution M1B anatomy, not patient-level M2 or M3.

S4.4 RGC studies

Current products vary from hESC- or iPSC-derived RGC-like cells and organoid-enriched precursors to directly induced neurons and scaffold-supported retinal neurons. They commonly express combinations of BRN3B, RBPMS, SNCG, THY1 and neuronal cytoskeletal markers, and several preparations fire action potentials or receive spontaneous postsynaptic currents in vitro [78–82]. These data can establish a credible M0 product. They do not show that the cells have acquired an adult RGC subtype, the correct dendritic stratification programme,

axon initial-segment organisation, sustained conduction capacity or the molecular guidance repertoire needed for a specific central target.

The phrase 'RGC-like' is therefore scientifically useful when competence remains partial. The mammalian retina contains numerous RGC subtypes with different receptive fields, firing properties, projection targets and vulnerability to disease. A bulk marker panel can confirm broad lineage while concealing subtype imbalance or developmental immaturity. Directly induced human cells, for example, resembled fetal rather than adult RGCs transcriptomically [83]. That immaturity may aid survival or growth, but it weakens any inference that generic RGC identity predicts appropriate adult connectivity. A replacement product should specify which visual functions and targets its subtype composition is intended to reconstruct.

The internal limiting membrane (ILM) and Müller-cell endfeet form the first major physical barrier after intravitreal delivery. Human stem-cell-derived RGCs placed on intact adult mouse retina largely remained superficial, whereas enzymatic or mechanical disruption increased somatic entry, topographic spacing and access to the ganglion-cell and inner-plexiform layers [84]. This establishes a strong barrier-mechanism claim and explains why cell dose alone has not solved integration. It does not establish functional replacement: improved access is M1B until donor physiology and host input are demonstrated.

Recent interventions refine rather than remove this trade-off. Low-dose plasmin increased the number of donor RGCs detected at one week, but the advantage was no longer evident at one month; higher-dose treatment impaired host pattern and full-field electroretinographic measures [85]. Focal laser photodisruption or collagenase treatment improved short-term survival or neurite entry in bovine and postmortem human explants, yet also injured endogenous RGCs or Müller endfeet and produced ectopic penetration beyond the intended layer [86]. These studies show that an intervention can improve the transplantation endpoint while worsening the tissue that must support it. Barrier modification must therefore be assessed simultaneously for donor access, host injury, inflammation, retinal physiology and durability.

Aguzzi and colleagues compared three ILM-disruption strategies across mice, rats, macaque tissue and human explants and found that disruption enabled donor entry into the ganglion-cell layer, dendritic extension into the inner plexiform layer and axon growth along the nerve-fibre layer towards the optic-nerve head [20]. The cross-species convergence makes the barrier conclusion compelling. Translation nonetheless remains non-trivial. A diseased human ILM may be thickened or surgically altered; a treatment field large enough to support useful vision may expose a correspondingly large area of host retina; and an intervention that removes Müller-endfoot integrity can change extracellular diffusion, trophic signalling and electrophysiology independently of the graft. The ILM procedure is part of the causal treatment package and requires its own matched control.

Across studies, the most reproducible in vivo achievement is sparse survival with variable retinal entry. Early hESC-derived RGC transplantation demonstrated short-term marker-positive cells after delivery but did not test mature donor physiology or central reconnection [78]. Organoid-derived populations survived for up to four weeks in only a subset of recipients, with marked attrition and little persistent neurite growth [79]. Human iPSC-RGC precursors treated with chondroitinase and RGC-like cells enriched from retinal organoids likewise provided human nuclear or lineage evidence of retinal residence and local processes over short intervals, but no donor-resolved afferent physiology [81, 82]. These are necessary M1 observations; describing them as functional engraftment would exceed the assays.

Longer follow-up does not by itself close the mechanistic gap. Mouse pluripotent-stem-cell-derived RGCs were still detectable at 12 months in two of four long-term recipients, but overall survival was at most approximately 5%, cellular overgrowth occurred, and axons entered the optic-nerve head mainly when donor somata lay nearby; many processes were short or directionally disorganised [87]. A biodegradable scaffold supported epiretinal human iPSC-derived retinal neurons and optic-nerve-directed projections in non-human primates for up to a year [88]. These studies materially inform persistence, delivery and safety. Without donor input physiology, long-distance tract reconstruction or central synaptic function, they remain M1A/M1B plus anatomical milestones towards M3-RGC-B.

Anatomical synapse claims need equal discipline. Developing or progenitor-derived RGCs have shown dendritic localisation and candidate contacts after transplantation [89]. Mouse ESC-derived RGC retinal fragments produced greater inner-plexiform integration in NMDA-injured than control retinas and showed presumptive

synaptic structures in selected tissue [90]. Apposition of synaptic markers, neurite interdigitation or even an ultrastructural specialisation can demonstrate that appropriate components assemble. It cannot show response sign, synaptic strength, receptive-field organisation or whether the contact contributes to a light-evoked spike. Such evidence strengthens M1B and the plausibility of M3-RGC-A; it is not physiological coupling by itself.

Material transfer is not confined to the outer retina. After ILM disruption and human RGC xenotransplantation, donor-derived cytoplasmic, nuclear-associated and mitochondrial labels were detected in host Müller glia [91]. Transfer was rare, but rarity is not the relevant threshold when the replacement claim may rest on a small number of labelled cells. The study establishes an alternative mechanism with high causal confidence; it does not imply that all reported donor RGCs are misidentified. It does mean that a transferable reporter cannot be the sole basis for donor identity precisely in the preparations in which the donor–host interface has been deliberately opened.

Most functional transplantation experiments use healthy retina, acute NMDA depletion or optic-nerve crush. Healthy retina supplies intact bipolar and amacrine partners and a permissive visual pathway; it tests access to a functioning circuit, not replacement in disease. NMDA injury rapidly removes inner-retinal neurons but does not reproduce decades of glaucomatous remodelling. Optic-nerve crush is valuable for neuroprotection, yet leaves the donor facing an acutely severed pathway rather than chronic pressure-related axonopathy. Evidence from these models should be described as mechanism-specific, not as a direct estimate of clinical efficacy.
